# Postglacial bioweathering, soil nutrient cycling, and podzolization from palaeometagenomics of plants, fungi, and bacteria

**DOI:** 10.1126/sciadv.adj5527

**Published:** 2025-05-07

**Authors:** Barbara von Hippel, Kathleen R. Stoof-Leichsenring, Uğur Çabuk, Sisi Liu, Martin Melles, Ulrike Herzschuh

**Affiliations:** ^1^ Alfred Wegener Institute, Helmholtz Centre for Polar and Marine Research, Polar Terrestrial Environmental Systems, Potsdam, Germany.; ^2^ Institute of Biochemistry and Biology, University of Potsdam, Potsdam, Germany.; ^3^ Institute of Geology and Mineralogy, University of Cologne, Cologne, Germany.; ^4^ Institute of Environmental Science and Geography, University of Potsdam, Potsdam, Germany.

## Abstract

Warming-induced glacier retreat exposes bare rocks and glacial sediments, facilitating the establishment of soils. The dynamic interplay between climate, vegetation cover, and soil formation is poorly understood as time-series data are lacking. Here, we present postglacial soil formation during the past 23,000 years inferred from ancient DNA shotgun analyses of Lake Lama sediments targeting plants, soil-associated fungi, and bacteria showing postmortem damage signatures that verify their ancient origin. In the Late Glacial, we reveal basaltic weathering with high abundances of lichens, carbon, and arsenic cyclers, shifting to mycorrhizae domination and N cycling in the Holocene. We reconstruct podzolization starting with spruce forest migration in the Holocene, resulting in soil acidification and increased iron cycling. Our reconstruction of soil formation also contributes basic knowledge for the design of carbon-capture strategies using basalt weathering.

## Teaser

From basalt weathering to podsolization, long-term soil development in Arctic tundra-forest recovered by palaeometagenomics.

## INTRODUCTION

Soils often feature as a static entity in terrestrial ecosystems, for example, in dynamic global vegetation models, despite it being known that they develop and even dynamically respond to drivers (*1*–*3*). This misconception originates, at least partly, from the lack of time series portraying the major soil processes including weathering (*4*), element cycling, and podzolization (*5*) and their reflection in soil communities (mainly plants, fungi, and microorganisms). For example, the initial soil establishment after deglaciation at the end of the last glacial and the subsequent soil development in response to climate-driven vegetation changes remain largely unexplored. However, understanding soil changes and their related drivers is necessary for decision-making to safeguard ecosystem services of soils including food production, forestry, and maintenance of ecosystem stability.

Pedogenesis is initialized by weathering of the parent material that is, among other processes, supported by plants, fungi, and bacteria (*6*–*8*). Lichens, as characteristic early colonizers, enhance weathering by using their hyphae to penetrate mineral cleavage planes (*9*) as well as releasing organic acids (*6*, *10*). Plant root exudates, for example low–molecular weight organic acids deriving from respiratory CO_2_ (*11*), additionally increase weathering. In more developed soils, ectomycorrhizae further enhance weathering when supplying plants with ammonium, resulting in an efflux of H^+^ and, subsequently, soil acidification (*12*). However, how basalt weathering changes on millennial timescales in relation to compositional changes of plants, fungi, and bacteria remains largely unexplored.

Nutrient cycling by fungi and bacteria in the rhizosphere, particularly of carbon, nitrogen, phosphorus, and sulfur, determines plant productivity, diversity, and composition (*13*). Most soil organic carbon originates from above- and belowground plant litter degradation and transformation (*14*). In addition to the plant-produced organic matter, soil organic carbon can be derived from atmospheric CO_2_ being fixed by multiple photo- and chemoautotrophic microbes in the soil (*15*), while heterotrophic bacteria degrade these fixed carbon compounds, later using them as a metabolic substrate, and releasing smaller parts as metabolites or as CO_2_ back into the atmosphere (*16*). Besides soil bacteria, saprotrophic fungi are also important for a first degradation of complex carbon compounds such as lignin (*17*). In the nitrogen cycle, N-fixing bacteria directly bind atmospheric nitrogen and convert it into a plant-available form (*18*). The plant uptake of nitrogen from the soil is supported by mycorrhizal fungi (*19*). Vegetation densification, such as forest establishment, results in an increased need for nutrient supply due to reduced turnover times of wood compared to soft tissue (*20*). Whether nutrient cycling is also changing on long timescales alongside soil development but with similar vegetation cover remains unknown. Also, whether a more complex nutrient demand in relation to vegetation densification results in a long-term diversification of the cycling pathways is still unknown.

Podzols are the common soil type in boreal forests that are typically dominated by *Larix*, *Picea*, or *Pinus* (*21*). These soils are characterized by low pH and show a high sensitivity toward further acidification due to low capacities for cation exchange and small amount of weatherable material (*22*). During podzolization, organic acids induce the release of aluminum and iron ions from rocks that then form chelates with organic matter (*21*, *23*). These complexes leach from the upper mineral horizons (bleaching) and become, at least partly, deposited in the subsoil, leading to its characteristic reddish-brown color (*21*, *23*). So far, podzolization has mainly been described along spatial gradients, and such studies do not help our understanding of podzolization temporalities. However, *Larix*-*Rhododendron* succession, for example, has been found to accelerate podzolization (*24*), although the specific and unique impact of ecological processes and environmental drivers are poorly understood. When podzolization started in the boreal forest and whether vegetation compositional changes can reverse podzolization processes remain largely unknown.

Directly assessing soil dynamics would greatly improve existing knowledge on soil development. Through erosion, soil-derived matter, including substantial amounts of DNA, can be transported into a lake (*25*). Consequently, the analysis of lake sedimentary ancient DNA (sedaDNA) has become a popular palaeoecological method (*26*). Hitherto, mostly metabarcoding approaches are applied to target single-organism groups including plants (*27*), or, rarely, fungi (*28*). Recently, metagenomic approaches emerged, enabling the study of complex ecosystems, for example, through sequencing the whole DNA contained in a sample (*29*, *30*). Obtained results were validated with modern studies (*31*) and traditional proxy data such as pollen, indicating that the sedaDNA signals contain reliable qualitative and quantitative ecological information (*27*, *32*–*34*). Such studies became possible because genome reference databases such as the widely used nucleotide database from National Center for Biotechnology Information (NCBI) (ftp://ftp.ncbi.nlm.nih.gov/blast/db/FASTA/nt.gz) have been markedly improved and extended recently. Despite the recent methodological improvements, palaeo-metagenomic studies targeting soil ecosystem development are hitherto entirely lacking.

Here, we show how postglacial soils became established and further developed in response to climate-driven vegetation change during the last about 23,000 years, using sedaDNA records of plants, and rhizosphere-related bacterial and fungal taxa from Lake Lama in north-central Siberia. An earlier manuscript version was published as part of a cumulative thesis (*35*). We show that the vegetation as well as temperature variation has an impact on the establishment of the soil microbiome, while time itself is less important. We also trace the weathering progress of the basaltic bedrock in the lake catchment, which shifted from a strong, lichen-dominated weathering during the Late Glacial to a generally weaker, mycorrhizae-dominated weathering in the Holocene. We also detected a turnover from carbon-dominated nutrient cycling during the Late Glacial to nitrogen-dominated cycling in the Holocene. Additionally, we reconstruct podzolization by showing increases in acidic-pH–preferring taxa in all the assessed subsets as well as rising iron cycling with mid-Holocene spruce forest expansion.

## RESULTS

### Compositional changes of plants, fungi, and bacteria in ancient metagenomic datasets

**Fig. 1.**
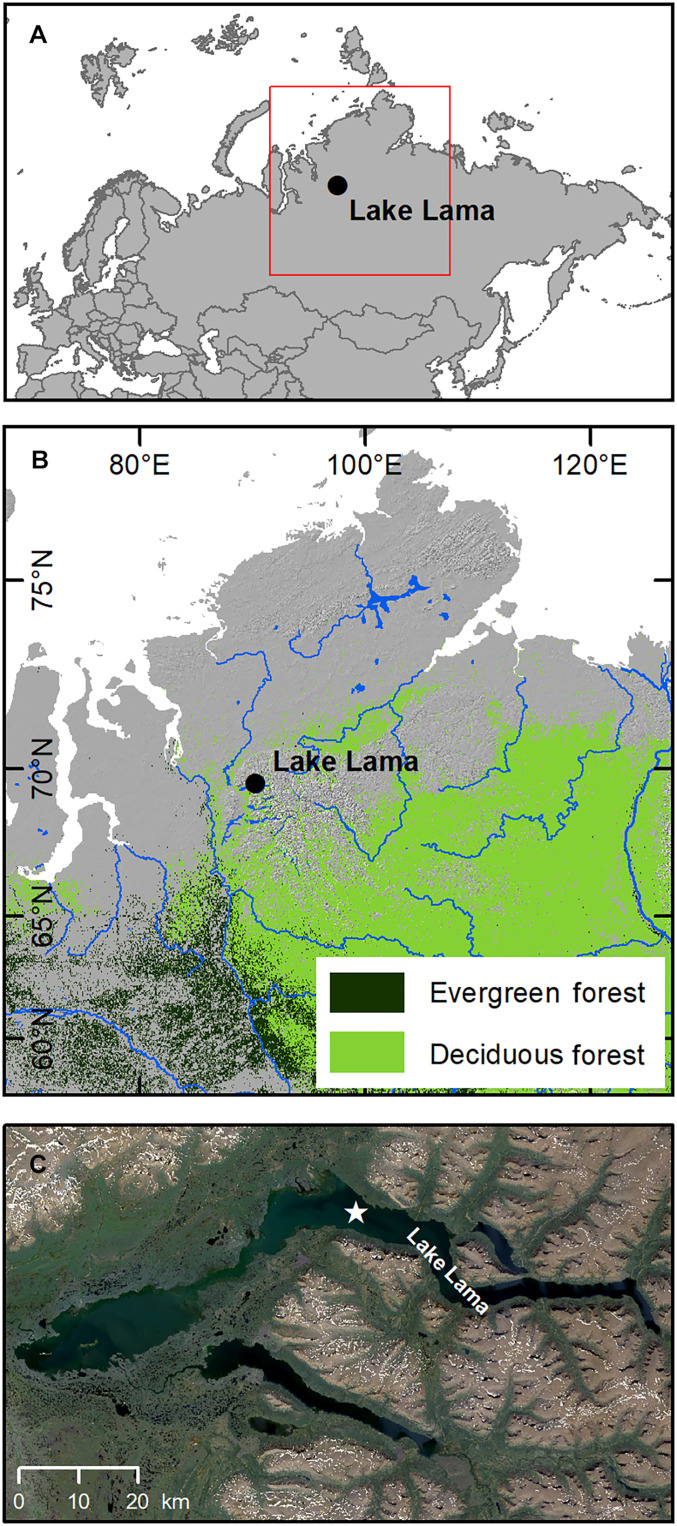
Overview map of the Lake Lama location. (**A** and **B**) show a map of central Russia and the study site. (**C**) Satellite image of the lake and its surroundings. The coring location in the lake is marked with a star. For the “distribution of deciduous and evergreen forests,” data from the ESA CCI Land Cover time-series v2.0.7 (1992–2015) dataset were used (www.esa-landcover-cci.org/). The land cover classes “70” (“Tree cover, needleleaved, evergreen”) and “80” (“Tree cover, needleleaved, deciduous”) were extracted for the illustration of the figure.

Shotgun sequencing data recovered from 42 sediment samples from Lake Lama (Fig. 1) yielded a total of 2,842,313,513 quality filtered reads, of which 21,593,970 (0.8%) were classified at least to root level by using Kraken2 against the nucleotide database. We used a strict confidence threshold of 0.8, providing precise classifications at the cost of a lower number of reads classified compared with a lower confidence threshold. Among the classified reads, 4,509,594 reads were assigned to bacteria with taxa assigned to genus or species level (20.9% from all classified reads), a subset of selected bacteria taxa (data S2) resulted in 3,998,339 reads (89% of all bacteria reads; fig. S4), which compose 1529 unique bacterial taxa. Among the classified reads 51,122 reads were assigned to fungi (0.24% from all classified reads), a subset of selected fungi taxa (data S2) resulted in 41,884 reads (82% of all fungi reads; fig. S4), comprising 1356 unique fungal taxa. Among the classified reads, 350,585 reads were assigned to Streptophyta with taxa assigned to genus or species level (1.6% from all classified reads), a subset of selected plant taxa (data S2) resulted in 259,136 reads (74% of all plant reads; fig. S4) including 304 unique plant taxa. Quality filtering of blank reads (six extraction blanks and 13 library blanks) yielded a total of 22,387,007 reads, which make up 0.9% of the total sequencing result of all samples and blanks together. Among the quality-filtered merged and paired reads, 22,215,852 (99.2%) were unclassified using Kraken2 against the nucleotide database with a confidence threshold of 0.8. Among the classified 10,985 reads (0.05% of all quality reads), only 2513 reads (0.01%) were assigned to the selected bacteria taxa, 118 reads (0.005%) were assigned to the selected fungi taxa, and 24 reads (0.0001072%) were assigned to the selected plant taxa.

Tests with less restrictive confidence thresholds 0.5 and 0.2 yielded significantly similar composition and temporal patterns as revealed by Procrustes analysis (table S1) and comparison of temporal trends of the major genera (fig. S5); however, we dedicated ourselves to use the more restrictive results (confidence 0.8) to minimize biases from false-positive taxonomic assignments. Moreover, tests comparing the taxonomic classifications from Kraken, HOPS, and HOLI yielded significant agreement in the temporal and compositional pattern and such confirm a low influence of classifiers and reference databases onto our findings (Supplementary Text, fig. S6, and tables S2 to S4).

Postmortem damage analyses of bacteria, fungi, and plant DNA in our dataset (see Materials and Methods and Supplementary Text) yielded a substantial share of reads that show an increase of C-to-T substitution toward the 5′ ends, i.e., the characteristic postmortem damage pattern of ancient reads (figs. S7A to S11A). Applying pyDamage to all taxonomic groups yielded an average C-T change of 0.22 for plants, 0.16 for bacteria, and 0.18 for fungi. Reads assigned to ancient contigs (prediction accuracy ≥ 0.6) were 59.7% for plants (1,557,792 reads), 79.3% for bacteria (399,002,412 reads), and 55.5% for fungi (5,918,560 reads). As a further confirmation of the ancient origin of the communities, we can show that the damage of the reads, exemplified by the share of C-to-T substitution rate at the first position, increases with age [figs. S7B, S10B, and S11B; Pearson correlation and *P* value for plants (HOPS): correlation coefficient (*r*) = 0.54, *P* < 0.001; bacteria (PyDamage): *r* = 0.16, *P* < 0.001; and fungi (PyDamage): *r* = 0.14, *P* < 0.001]. However, plant damage patterns from pyDamage were less reliable due to fewer reads/contigs, especially during the late Holocene (fig. S9). Despite this, damage patterns were evident across all taxonomic groups, although taxon-specific patterns were beyond the study’s scope.

We also show that the composition and turnover of the assemblage filtered for damage reads is similar to non-filtered assemblages (Supplementary Text, figs. S6 and S12, and table S5). From these results, we infer that the temporal compositional pattern of plants, fungi, and bacteria that we interpret with respect to soil development originates from ancient palaeogenomic signals, not mainly from post-sedimentary or post-sampling compositional changes (further evaluation in Supplementary Text).

**Fig. 2.**
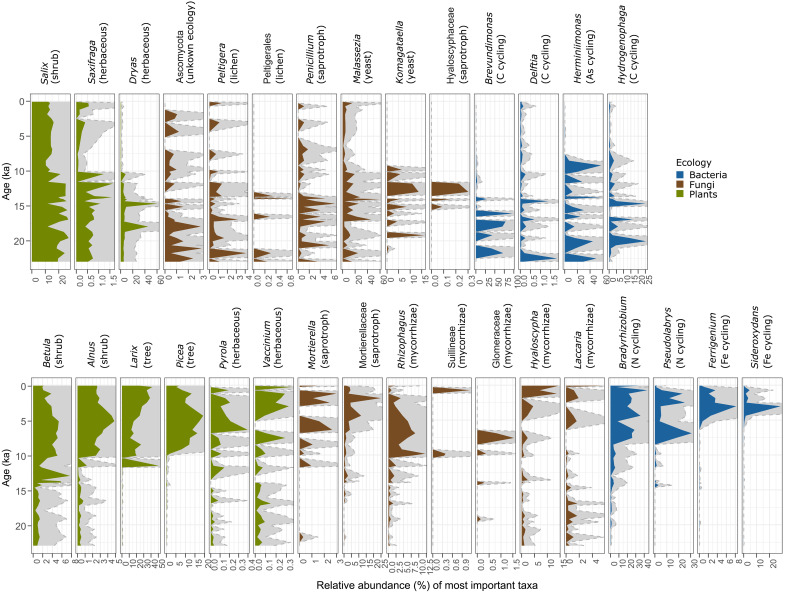
Relative abundance of the most prominent plants, fungal, and bacterial taxa recovered from the sediment of Lake Lama. The abundance is relative to the taxa recovered in the respective subset (plants, fungi, and bacteria). In brackets is the respective vegetation type, fungal ecology, or bacterial element cycle. (**A**) Most prominent taxa in the Late Glacial. (**B**) Most prominent taxa in the Holocene.

**Fig. 3.**
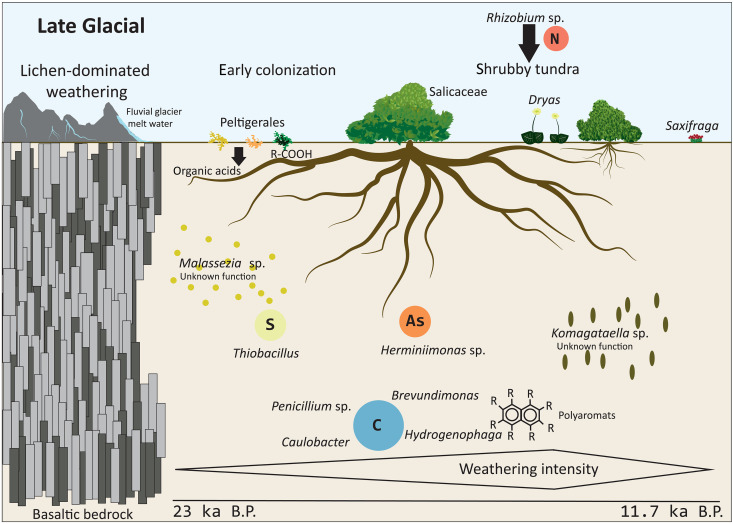
Ecosystem and soil dynamic changes over time. Top part: Development and changes in the ecosystem over time during the Late Glacial, when glacial melt-water flow and lichen cover lead to strong weathering of the basaltic bedrock. After early colonization, the vegetation subsequently developed toward shrubby tundra. Bottom part: Carbon (C) cycling in the soil was high. Sulfur (S) cycling was high during early colonization, while arsenic (As) cycling was prominent until the onset of the Holocene. The size of the circles represents the importance of the respective element cycling process. All cyclers besides *Penicillium* (fungus) are representing bacterial taxa. Yeast taxa were highly abundant throughout the Late Glacial.

**Fig. 4.**
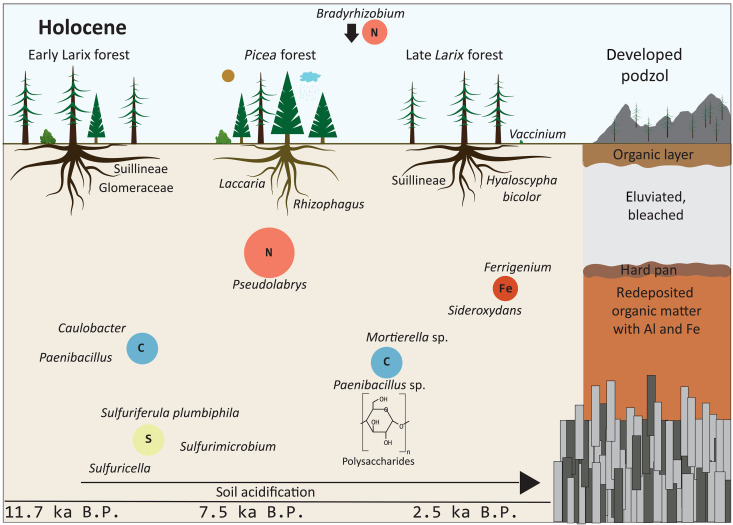
Soil establishment during the Holocene, resulting in the formation of podzol. With the onset of the Holocene, Pinaceae invaded the area and an early *Larix* (larch) forest was established. During the thermal optimum in the Holocene, this early *Larix* forest was replaced by *Picea* (spruce), leading to an increase in iron (Fe) cycling in the soil and the start of podzol development. The changes in the soil properties enabled the remigration of *Larix* with *Vaccinium* taxa as an herbaceous soil cover. The nutrient cycling in the Holocene is dominated by nitrogen (N) cyclers in the soil. Additionally, carbon (C) cycling changed toward polysaccharide cycling and sulfur cycling diversified. In the late Holocene, iron cycling increased. All cyclers besides *Mortierella* (fungus) are representing bacterial taxa.

There is a median of 385 plant reads per sample, with 304 plant taxa identified (*35*). Among them, 44.8% of the reads are assigned to species level, while 55.3% are assigned to genus level. The vegetation shows overall compositional change from tundra-dominated Late Glacial to taiga-dominated Holocene, underlining studies from pollen and sedimentary ancient DNA (sedaDNA) data from other sites in northern Siberia [e.g., (*36*, *37*)]. The Late Glacial is characterized by a typical glacial flora with high abundances of *Dryas* (avens) and Saxifragaceae as well as *Salix* (willow) in the river valleys (Figs. 2 and 3). After about 14 thousand years (ka), *Betula* (birch) expands, and, with the onset of the Holocene, *Alnus* (alder) and Pinaceae markedly increase in the record. For the early Holocene, the data show a massive expansion of *Larix* (larch), followed by *Picea* (spruce) during the mid-Holocene and a readvance of *Larix* during the late Holocene (Figs. 2 and 4). With the arrival of *Picea*, the herbal community changes: Galegeae is absent from thereon and also *Dryas* (avens) decreases in abundance, while Ericaceae, including *Pyrola rotundifolia* (round-leaved wintergreen), and Asteraceae increase in association with the *Picea* forests.

There are 1356 unique fungal assignments, with a median of 302 reads per sample (*35*). Among the fungal reads, 34.3% are assigned to species level, 23.9% to genus level, and 14.31% to family level. The remaining 27.6% are at class level or above. We find a general trend from an Ascomycota-dominated Late Glacial with a relatively high abundance and diversity of lichens to a Basidiomycota-dominated Holocene (many of them known as mycorrhiza-forming taxa), matching the spatial gradient observed in glacier forefields (*38*). Modern studies on tundra and taiga soils from the Kola Peninsula show a *Penicillium* dominance in both biomes (*39*), which is contrary to our findings: Saprotrophs show a shift from *Penicillium* dominance during the Late Glacial toward *Mortierella* species in the late Holocene in our record (Figs. 2, 3, and 4). We found an overall high abundance of yeast taxa in the Late Glacial (*Malassezia* spp. and *Komagataella* spp.). Similarly to yeast, lichens (Peltigerales, *Peltigera* spp.) are more abundant in the Late Glacial than in the Holocene. Our data indicate that mycorrhizal taxa (Suillineae, Glomeraceae, *Rhizophagus*, *Laccaria*, *Hyaloscypha*, and Tuberaceae) gained in importance with warming at the onset of the Holocene.

We recovered 1529 bacterial assignments at genus and species levels with a median of 26,628 reads per sample (*35*). We restricted the analyses to taxa with known occurrences in soil (see Materials and Methods). Among them, we recovered 65% reads at species level and 35% at genus level. Like with the vegetation and fungi, the major compositional shift for bacteria occurred at the Late Glacial–Holocene transition (Figs. 2, 3, and 4). We discovered *Brevundimonas* and *Hydrogenophaga* (both involved in carbon-cycling genera) mainly in the Late Glacial. Additionally, arsenic cyclers from the genus *Herminiimonas*, which oxidize arsenite, are highly abundant throughout the Late Glacial (*40*). In contrast, *Bradyrhizobium* (nitrogen fixation), *Ferrigenium* (iron oxidation), *Sideroxydans* (iron oxidation), and *Pseudolabrys* (ammonia oxidation) show high abundance in the Holocene samples.

## DISCUSSION

### Long-term soil development: A trajectory or environmentally driven processes?

The time-series data on soil fungi and bacterial community changes trace (*35*) long-term postglacial soil development that has hitherto only been investigated along spatial gradients [e.g., (*38*, *41*)]. Previous aDNA shotgun studies have focused on changes in aboveground terrestrial ecosystems [e.g., (*27*)], lakes (*42*), or oceans (*43*). Our analysis of the postmortem damage patterns indicates that a significant portion of the analyzed plant, fungal, and bacterial reads likely has an ancient origin (Supplementary Text). The temporal consistency between pollen data (*37*) and sedaDNA, as well as the agreement among the three different sedaDNA-based taxa group signals, further corroborates this conclusion. Nevertheless, substantial knowledge gaps remain regarding taxa-specific DNA preservation (*44*) and variations in the proportion of ancient communities across taxonomic groups (*45*, *46*), necessitating further investigation.

**Fig. 5.**
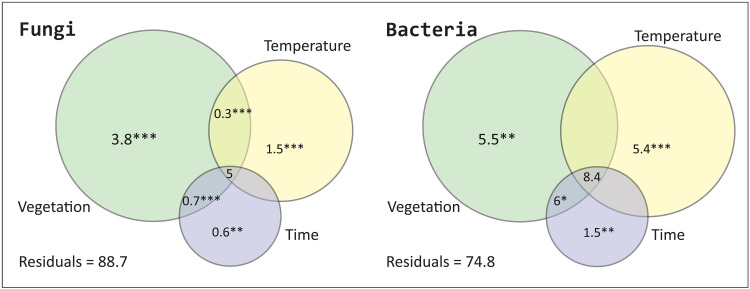
Variation partitioning of fungi and soil bacteria. The numbers are the percentage of explained variation for the respective driver (for overlapping areas the combined variation). We show that vegetation as a single variable has the greatest impact on the establishment of either community, followed by the temperature. The unexplained variation is given as the percentage of the residuals. The asterisks indicate the statistical significance of each single result (*P ≤ 0.05; **P ≤ 0.01; ***P ≤ 0.001). Values < 0 are not shown.

From variation partitioning using constrained ordination (Fig. 5) (*35*), vegetation explains the highest unique amount of variance in the fungal compositional data, followed by temperature (see Materials and Methods), while time passed since deglaciation uniquely explains only a minor variation in the dataset. Similarly, vegetation and temperature uniquely explain a relatively high amount of variation in the bacterial dataset.

Generally, our findings (*35*) on the importance of vegetation and temperature on soil development confirm spatial and experimental studies. For example, the rapid migration of *Betula nana* (dwarf birch) in the Arctic tundra has been identified as a main driver of soil microbial shifts after experimental warming (*47*). Also, *Alnus* (alder) has been found to affect the establishment of bacterial communities after glacier retreat (*48*). In tundra communities from the Taymyr Peninsula, vegetation cover also highly affects the composition and biomass of fungi and bacteria (*49*). We find that temperature has a greater impact on the bacterial community than on fungal composition, which is in contrast to experimental evidence from a pine forest (*50*).

Overall, our results indicate that postglacial soil development on a millennial timescale represents environmentally driven processes rather than a pure trajectory, that is, time passed since glacial retreat explains only a small unique variance in the bacterial and fungal compositional changes (*35*). This agrees with the conclusion of Delgado-Baquerizo *et al.* (*3*) who compared multiple topsoils worldwide of varying ages and showed that parent material, climate, vegetation, and topography have a much greater impact on soil development than soil age has. However, our results disagree with the finding that time since recent deglaciation is most important for soil microbiome establishment (*51*). Most of the variance in our fungal and bacterial data is not explained at all, and most of the variance is explained by a combination of tested variables, indicating that we may have missed major drivers and/or internal dynamics [e.g., external nutrient supply (*52*) and variation in wetness (*53*)] and that soil-temporal-environmental relationships are complex. We also need to consider that DNA preservation varies across sedimentary layers due to differences in taphonomic processes.

### Bioweathering supported by lichens and mycorrhiza

**Fig. 6.**
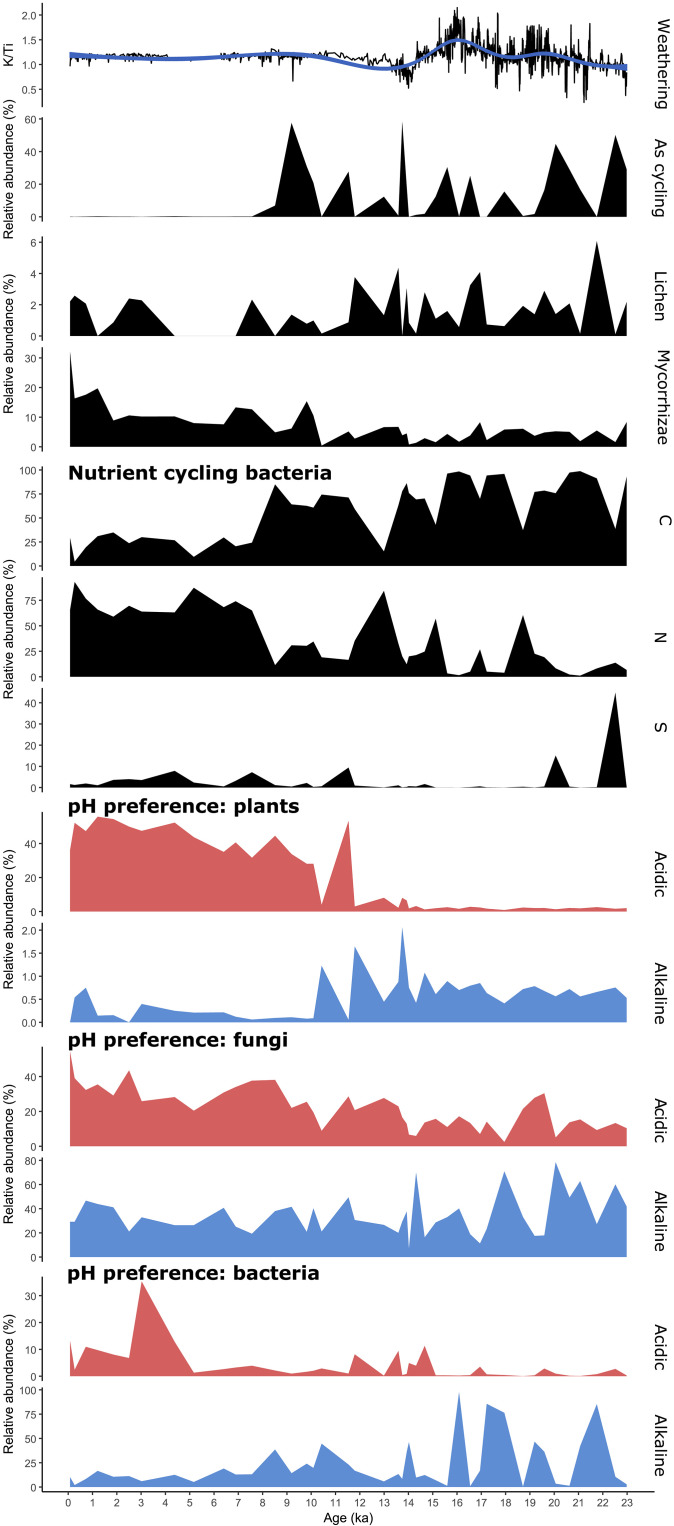
Reconstruction of soil development from Lake Lama sediment. General weathering is shown by the K/Ti ratio of the XRF data. Arsenic cycling bacteria are prominent in the samples from the Late Glacial up until 7.5 cal ka B.P. A transition in relative abundance from lichen toward mycorrhizae is apparent throughout the core. We display a turnover in nutrient cycling bacteria from carbon (C)–dominated cycling in the Late Glacial to nitrogen (N)–dominated cycling in the Holocene and show the sulfur (S) cycling alongside. The pH preferences (acidic/alkaline) of the taxa of the respective subsets show an acidification with increasing soil age and ongoing development.

Basalt, forming the bedrock in the Lake Lama catchment, is largely composed of feldspar silicates containing high amounts of potassium, which is a mobile element released through weathering (*54*). Thus, we (*35*) interpret a high ratio of mobile K to immobile Ti (Fig. 6) in the sediment as a proxy for strong soil weathering (*55*).

We reveal enhanced weathering during the initial phase of soil formation shortly after deglaciation (*35*). According to the fungal record, this is at least partially related to lichens (and their release of organic acids) that are known to be early colonizers of basaltic rocks (*9*, *56*). A second phase of high weathering occurred during the phase of maximum *Salix* abundance when glacial meltwater percolated the soils during the Bølling/Allerød warm period (Figs. 2 and 6).

Strong basalt weathering in the Late Glacial is also confirmed by the high abundance of arsenite oxidizers (mainly *Herminiimonas arsenicoxydans* and *Herminiimonas arsenitoxidans*; Figs. 2 and 6) in our bacterial record. It is likely that the weathering of basalt led to the release of iron hydroxides (*57*), which have a high affinity to bind to arsenic (*58*). Arsenic-cycling taxa oxidize the arsenic anion to arsenate (*40*), a process that is highly needed after the high arsenic release from initial rock weathering.

Weathering declined with *Larix* forest expansion after the onset of the Holocene, alongside a decline in lichen and an increase in mycorrhiza relative abundance (Figs. 2 and 6) (*35*). Previous short-term studies have demonstrated a decline in lichen abundance and diversity with warming (*59*). This indicates that lichens in the catchment acted as the main primary rock weathering fungi for the initial rock breakdown (*56*), while mycorrhizae took over the role for finer mineral weathering by releasing inorganic nutrients from minerals after first soil establishment (*60*). Warming is assumed to affect the diversity and composition of mycorrhizal communities rather than their relative abundance (*61*), an assumption supported by our data.

On the taxon level, we find an increase in mycorrhizal *Rhizophagus* as well as Glomerales with the onset of the Holocene (Fig. 2) (*35*). Glomerales are arbuscular mycorrhizal fungi, living in symbiosis with around 80% of the vascular land plants (*62*). Our data suggest a strong dependency of woody taxa on arbuscular mycorrhizae compared to tundra species (Figs. 2 and 6). Suillineae co-occurred with *Larix* migration at the onset of the Holocene, disappeared during the *Picea* forest stage, and reappeared in the late Holocene with a second peak in *Larix*, as confirmed by co-occurrence analysis (Fig. 2 and fig. S3). This finding supports studies by Zhou and Hogetsu (*63*) and Praeg and Illmer (*64*), who highlight Suillineae as an important *Larix* mycorrhizal associate. Suillineae species are ectomycorrhizal fungi, usually a symbiosis of woody taxa and Asco- or Basidiomycetes (*21*), which are known to enhance weathering by secreting oxalate (*65*).

We also note an increase in *Laccaria* species after 5 calibrated thousand years before the present (cal ka B.P.) (Fig. 2) (*35*). *Laccaria* are known to form mycorrhizae with not only Pinaceae but also Salicaceae and Fabaceae (*66*). In mycorrhizal associations with *Larix*, *Laccaria* is known to reduce the amount of phenolics, which defend plant roots against parasitic fungi (*67*). In the late Holocene, *Hyaloscypha* species became more abundant, coinciding with increasing *Vaccinium* abundance (blueberry; the family Ericaceae), confirming known interactions from modern studies (*68*). Overall, most of our detected mycorrhizal taxa are known to be nonspecific to distinct plant species (*62*). This suggests that mycorrhizal fungi in such an extremely cold and nutrient-poor habitat must be generalists, supporting a broad diversity of plants in their growth.

### Turnover in carbon, nitrogen, and sulfur cycling

Our bacterial record reveals a dominance of carbon cyclers during the Late Glacial tundra phase, while nitrogen cyclers become more abundant with woody-taxa densification from about 15 ka on (Fig. 6) (*35*). This finding supports a 7-year monitoring study on the modern Siberian tundra-taiga ecotone that shows increasing nitrogen cycling with densification of the tree stands (*69*). Additionally, the N content in tundra soils was generally increased when exposed to warming (*70*). We find the major turnover from carbon to nitrogen cyclers occurred at the transition from light *Larix*-dominated forest to dark *Picea*-dominated coniferous taiga. Unexpectedly, our study suggests that the forest composition has a larger effect on C/N cycling than the general rapid migration of forest.

As well as their relative share, the composition of the carbon-cycling community also shifted along with vegetation change (Figs. 2 and 6) (*35*). While we recovered a high relative abundance of polyaromatic degraders in the Late Glacial (*Brevundimonas* and *Caulobacter*), polysaccharide degraders gained importance in the Holocene (e.g., *Paenibacillus* spp.). This aligns with modern spatial gradients where tundra soils in northern Siberia were found to contain polyaromatic compounds (*71*), while forest soils generally had a larger proportion of microbial polysaccharides (*72*).

A shift from bacterial-only carbon cycling to fungal-bacterial co-cycling is revealed (*35*), probably because the boreal litter is very difficult to decompose due to its high amount of phenol-rich substrates (*73*). It is known that the increasing abundance of phenolic acids in soil has a stimulating effect on the abundance of saprotrophic fungi (*74*) as they can tolerate high concentrations of phenolic compounds and degrade them (*75*).

We also detect a diversification of the nitrogen pathways (Figs. 2, 4, and 6) (*35*). During the Late Glacial, nitrogen fixation was mainly from the air (*Rhizobium* spp.). Wettening and warming in the Holocene (*37*) and subsequent establishment of dark taiga resulted in a diversification of nitrification processes, including ammonium oxidation (*Nitrosomonas* spp.) as well as nitrite oxidation (*Nitrotoga* and *Pseudolabrys*). To date, current knowledge diverges: Some studies showed that increasing moisture (*76*) and temperature, to a certain extent (*77*), stimulate nitrogen fixation in high Arctic ecosystems (*78*). Experimental warming can also induce a decline in nitrogen fixation in Arctic tundra sites (*76*). Previous studies (*79*, *80*) have shown that, depending on the tree species, between 20 and 40% (*Picea* plantation) and 70% (*Picea abies* forest) of the nitrogen fixation from the air is retained in the canopy. Our study suggests that the establishment of (dark) evergreen taiga results in higher foliage retention, meaning that nitrogen is directly captured in the crowns without microbial biomass being involved (*81*). Subsequently, the fixation of nitrogen in soil from the air is hindered (*82*), and the nitrogen cycling pathways diverge.

Sulfur cyclers show a peak dominated by *Thiobacillus* during the initial plant establishment when sulfur cycling was vital for amino acids (methionine and cysteine) and such protein synthesis (Figs. 2 and 6) (*35*). In contrast, we assume that low S cycling occurs during phases of increased weathering. During these phases, S adsorbed onto Fe and Al hydroxides likely became a good source of plant-available sulfur as well as a hindering S leaching (*83*) such that the need for additional bacterial cycling was low. A warmer climate and the establishment of forest soils resulted in stronger sulfur cycling, as indicated by high abundances of sulfur oxidizers, including *Sulfuriferula plumbiphila*, mostly replacing *Thiobacillus*. Warming has previously been demonstrated to result in a higher relative abundance of sulfur cycling genes in tundra soils (*84*) and to induce high amino acid turnover (mineralization and, subsequently, reuptake) in Alaskan taiga soils (*85*). Our data underline the demand for diversified sulfur sources with slower plant turnover in boreal forests.

### Tracing podzolization

We reveal an increase in taxa preferring acidic soil alongside soil development (Fig. 6) (*35*). The peak of acidophilus plants is observed at 2.5 cal ka B.P. when they make up around 50% of the relative abundance (mainly spruce, larch, alder, birch, pine, and blueberry; *Picea*, *Larix*, *Alnus*, *Betula*, *Pinus*, and *Vaccinium*) of taxa with known pH preferences. This agrees with modern data comparing multiple forest sites, which show that *Picea* forests have the lowest pH (*86*). Slowly decomposing litter (*87*) as well as poor buffer capacity (*88*) in evergreen *Picea* forests lead to recalcitrance [e.g., high C/N ratios and lignin concentrations; (*87*)], while *Larix* litter has comparatively high base cation fluxes (*89*). Additionally, less nutrients in the soils in *Picea* forest inhibit organic matter breakdown, leading to the formation of organic acids and subsequently to acidification of the soil (*21*, *23*).

The bacterial community also shows a strong peak in acidophilus taxa in the late Holocene (Fig. 6) (*35*). The impact of soil acidification on the composition of bacterial communities is known to be driven by ecological filtering (i.e., better adapted taxa invade in the area) (*90*). The acidic-preferring bacteria in our data show a shift from mainly *Delftia* species during the Late Glacial toward *Sideroxydans* dominance in the Holocene.

Fungi also show an increasing acid preference in the late Holocene (*35*), although the signal is not as pronounced as for the other organismic groups, being underlined by many acid-tolerant fungi such as *Trichoderma* or *Hyaloscypha*, which occur throughout the whole record (Figs. 2 and 6). Rousk and Bååth (*91*) find that lowering of the pH generally leads to increased fungal growth, while bacterial growth is decreased. Our data take this further by showing that lowering of the pH leads to more acid-tolerant bacteria, while fungal communities are less affected by pH changes. Alongside the acidification of the soil, we see evidence for podzolization with increasing iron cycling around 7 to 6 cal ka B.P. in our record (Figs. 2 and 6), coincident with the onset of the temperature and moisture maximum in the region (*37*), suggesting that podzolization is, to some extent, also warmth and rain induced.

The strong peak in iron-cycling bacteria (mainly *Ferrigenium kumadai* and *Sideroxydans*) in the late Holocene (*35*), alongside the re-expansion of *Larix* into *Picea* forests, indicates an iron deficiency induced by leaching (Figs. 2 and 6). We further detect an increased relative abundance of *Vaccinium* in the late Holocene, suggesting compositional differences between the early *Larix* forest at the onset of the Holocene and the late Holocene *Larix* forest that resulted from podzolization during the preceding *Picea* phase. A comparison of modern *Larix* and *Picea* communities reveals a higher Fe concentration in the soil with *Picea* than in that with *Larix* (*92*). We assume that the iron cycling is not affected by the general presence of *Larix*, but the change in soil composition induced by the preceding *Picea* is evidence of a trajectory of soil development in the area.

### Implications and conclusions

By analyzing sedaDNA shotgun metagenomics from sediments from Lake Lama (northern-central Siberia), we reveal a pronounced vegetation shift from tundra toward taiga at the Late Glacial–Holocene transition (*35*). Alongside, we find a lichen decline but an increase in mycorrhizae as well as a shift from carbon-dominated nutrient cycling toward nitrogen dominance.

With our study, we have shown that lake sedaDNA not only is a valuable tool for analyzing compositional changes of plants, fungi, and bacteria but also allows the reconstruction of soil development. While our postmortem damage analyses confirm that the compositional signal is mainly of ancient origin, in future studies, biases from post-sedimentary and post-sampling compositional changes require more attention.

We show that the establishment of fungal and bacterial soil communities is, to a great extent, influenced by the vegetation cover, followed by temperature variation, while the time since initial soil development only plays a minor role (*35*). This suggests that there could be significant turnover in the soil microbiome under future global warming alongside shifting treelines. As the relationship between plants and their associated microorganisms is rather tight, understanding drivers of soil microbiome communities is an asset when developing advanced fertilizers adapted to global warming scenarios.

We found evidence of rapid initial weathering of basalt after glacier retreat with herb- or shrub-dominated tundra, which declined with the warming-induced taiga advance (*35*). Understanding past weathering enables the application of its mechanisms to address ongoing global challenges: Weathering of basalt is a known carbon sink for atmospheric CO_2_ (*93*, *94*). Powdered basalt grains can be applied to soils to enhance weathering and thus lock-up large amounts of carbon dioxide, removing it from the global carbon cycle (*95*). We show that the most basalt weathering occurred during *Salix* dominance in the river valleys, which is of particular interest when assessing general soil development under ongoing global change and making use of basaltic carbon capturing potentials.

We could show a shift from carbon-dominated nutrient cycling in the Late Glacial toward intensified nitrogen cycling in the Holocene (*35*). An intensified need for diverse nitrogen cycling in taiga vegetation in comparison to tundra is noted, highlighting the differences between tundra and taiga turnover times. Our data additionally reveal a diversification of sulfur sources in boreal forests for plant establishment and their amino acid synthesis. Together, the data provide strong evidence for a relationship between nutrient pools and cycling in relation to plant life cycles.

Last, our data also trace the establishment of podzol in the study area with increasing iron cycling in the Holocene as well as soil acidification (*35*). We highlight that the early and the late larch forests show differences in their underlying herbaceous taxa, inferred from changing soil and, therefore, growing conditions. The podzolization process was initiated with the establishment of dark evergreen taiga. The remigration of larch forest into the area in the late Holocene indicates that soil establishment is a trajectory as podzolization is irreversible despite changing vegetation cover. This might be an important result for forecasting future plant establishment or even be applicable to foster soil development in agricultural settings where multiple plant types need to be grown on the same ground.

Our study (*35*) provides basic knowledge for understanding and forecasting treeline advance under future global warming. It forms the base for the development of potential afforestation strategies and as such highlights the potential of large-scale carbon-capture enhancement through boreal forest establishment alongside basalt grain weathering.

## MATERIALS AND METHODS

This study presents results earlier included in a cumulative thesis (*35*).

### Geographical setting and study site

Lake Lama (69.32°N, 90.12°E; 53 m above sea level) is located on the Taymyr Peninsula, northern-central Siberia, at the western rim of the basaltic Putorana Plateau (Fig. 1). The current vegetation in the area consists of dense taiga with *Picea*, *Larix*, and *Betula*, as well as shrubs such as *Alnus fructicosa*, *Salix*, and *Juniperus communis* and dwarf shrubs (*37*). Modern mean temperatures are 13.8°C for July and −28.8°C for January [Volochanka weather station; distance to the lake, 247 km; (*96*)]. In 1997, an 18.85-m-long sediment core (PG1341) was retrieved from the lake at a depth of 66 m, dating back to the last about 23 ka. Before processing, the sediment has been stored in the dark and at 4°C. We refined the age-depth model of von Hippel *et al.* (*36*) (fig. S1).

### XRF scanning of the sediment core

X-ray fluorescence (XRF) scanning (*35*) was conducted at the University of Cologne, Germany, on one core half using an Itrax core scanner (Cox Analytical Systems, Sweden) equipped with a Cr tube and a silicon-drift detector in combination with a multichannel analyzer. Analyses were performed at 30 kV and 55 mA, at a resolution of 2 mm and an integration time of 6 s. Results are semiquantitative estimates of relative concentrations of the detected elements (*97*), derived from the detected peak area intensities and given in total counts per second. The K and Ti count data were normalized to the K/Ti element ratio to account for variations in organic content and other elements (*98*).

### Core subsampling

Before subsampling, the cores were split in half (*35*). One half was stored as the archive half, and the other one was used as work half. The sub-core segments were sampled for sedaDNA in the climate chamber of the Helmholtz Centre Potsdam - German Research Centre for Geosciences (GFZ) remote from any genetic laboratory. The chamber was cleaned with DNA Exitus and deionized water before sampling. All sampling devices were sterilized with DNA Exitus and ultraviolet light radiation for 1 hour before usage. During the sampling process, protective clothing as well as face masks and hair nets were worn. Before sampling, the surfaces of the cores were scraped twice with clean knives that were treated with DNA Exitus and deionized water. The samples were taken using four knives while only sampling the interior section of the work half of the core to avoid contamination with modern bacteria. The samples were then placed in sterile 8-ml Sarstedt tubes and frozen to −20°C until further processing. We included a total of 42 samples in the study, with an interval of ~500 years between samples.

### DNA extraction

The extraction of the sedaDNA (*35*) was conducted in the dedicated aDNA laboratories at Alfred Wegener Institute, Helmholtz Centre for Polar and Marine Research (AWI), Potsdam, using the DNeasy PowerMax Soil DNA Isolation Kit (QIAGEN), following the manufacturer’s instruction. An additional incubation step overnight in a rotation incubator at 56°C was added, and the elution of the DNA was performed as described in von Hippel *et al.* (*36*). After the extraction, the DNA was concentrated using the GeneJET PCR purification Kit (Thermo Fisher Scientific, Germany) by which 1 ml of the DNA extract was reduced to a volume of 50 μl. The concentrated extracts were measured with a Qubit dsDNA BR assay kit using a Qubit 4.0 Fluorometer (Thermo Fisher Scientific, Germany), diluted to a final concentration of 3 ng/μl, and stored in aliquots of 15 μl to avoid extensive freeze-thaw cycles.

### Single-stranded DNA library build

The DNA libraries (*35*) were built following the single-stranded DNA library preparation protocol of Gansauge *et al.* (*99*) with the ligation of the second adapter in a rotating incubator as described by Schulte *et al.* (*100*), using 30 ng of DNA as input. Furthermore, the libraries were quantified with quantitative polymerase chain reaction (qPCR). Further details on the protocol are described by Schulte *et al.* (*100*). For the setup of the index polymerase chain reaction (PCR), we used 1× AccuPrime Pfx reaction mix, AccuPrime Pfx Polymerase (2.5 U/μl), 4 μl of P7_X indexing primer (10 μM) and P5_X indexing primer (10 μM), and 57 μl of deionized water. The final DNA library (24 μl) was added to the reaction. The PCR was conducted according to the following protocol: 2 min at 95°C, 20 s at 95°C, 30 s at 60°C, 1 min at 68°C, and final elongation for 5 min at 68°C. The appropriate number of amplification cycles (steps 2 to 4) for the index PCR was calculated from the qPCR results and varied between 11 and 13 cycles for samples and blank controls.

The PCR products were purified with MinElute (QIAGEN, Switzerland) according to the manufacturer’s instructions and eluted in 30 μl of elution buffer. The DNA library concentration was determined using a Qubit 4.0 Fluorometer dsDNA BR assay kit (Thermo Fisher Scientific, Germany). For the quality control and to measure the fragment length composition, we loaded the libraries on a TapeStation (Agilent, United States). Mean fragment length and concentration of indexed libraries were used to calculate the molarity of each library, and equimolar library pools were prepared. In total, we compiled four library pools.

The library pools APMG-37 (10 samples, four library blanks, and one extraction blank) and APMG-38 (10 samples, three library blanks, and two extraction blanks) were sent to Fasteris SA, Switzerland, and were run on a NovaSeq device [2 × 100 base pairs (bp)]. Each extraction batch includes nine samples and one extraction blank, while a library batch includes seven samples or extraction blanks and one additional library blank. A table with the sample composition of the sequencing runs as well as their metadata is provided in data S1. The third (23 samples, six library blanks, and three extraction blanks) and fourth (15 samples, whereof 14 were sample replicates to increase read counts for poorly sequenced samples) library pools were sequenced on an NextSeq 2000 platform (2 × 100 bp) at AWI Bremerhaven, Germany.

### Bioinformatic pipeline for the analysis of the sequencing results

The analysis of the raw sequencing data included a quality check using fastQC [version 0.11; (*101*)] and a deduplication step (removing identical reads) with clumpify (BBmap version 38.87, https://sourceforge.net/projects/bbmap/). The paired-end forward and reverse reads were merged with fastp [version 0.20.1; (*102*)] applying a low complexity filter to remove reads of low complexity from the dataset. Taxonomic classification was done with Kraken2 (*103*) against the nucleotide database by NCBI to yield a better comparable pipeline for all assessed taxa, so none of the selected groups becomes over- or underrepresented (ftp://ftp.ncbi.nlm.nih.gov/blast/db/FASTA/nt.gz; download: October 2022, with default *k*-mer size of 35). We prefer the usage of the widely used and resource efficient Kraken2 tool over other less widely used and resource-intensive tools to allow for a better reproducibility of our results by other groups. However, our comparison of the results gained by Kraken2 with all other pipelines now used to palaeometagenomic data including HOPS and HOLI yielded very similar compositional and temporal patterns (fig. S5 and tables S2 to S4). We applied a conservative confidence threshold of 0.8 (*100*). Additionally, we tested thresholds of 0.2 and 0.5 to assess the comparability between stratigraphic plots of the most prominent taxa for plants, bacteria, and fungi with less conservative thresholds (fig. S6 and table S1).

### Data analysis

The analysis of the processed DNA data was done in R, version 4.0.3. As a first step, we combined the converted kraken report file, with metadata (depth and age of the sediment samples) and a lineage file, which adds the full taxonomic lineage of the identified taxa via TaxID. The raw reads of all three sequencing runs were lastly merged in R.

Three taxonomic data subsets (plants, fungi, and bacteria) were created. For the plant dataset, we extracted all reads assigned to Streptophyta and kept those reads that were at least assigned to genus level. We cleaned the plant subset from aquatic and non-Siberian taxa (namely, taxa not occurring in a vicinity of 1000 km to the lake as identified using gbif.org). The fungi subset is defined by all reads of the kingdom “fungi.” Due to generally poorly sequenced fungal genomes and, therefore, their absence in databases, we decided to work on taxa assigned to at least phylum level for terrestrial fungi. The bacteria subset contains reads assigned to the domain of “bacteria.” Among bacteria, we only kept reads that were assigned to at least genus level. For microorganisms, we filtered for all taxa known from soil habitats and excluded taxa, e.g., characteristic for aquatic habitats. For all aquatic fungi and bacteria, we provide references to prove the selection criteria. We also verified the bacterial assignments using a scraping script with Python, taking BacDive as the reference database. After filtering, we keep 74% of all Streptophyta reads, 82% of all fungi reads, and 88% of all bacteria reads (fig. S4). The taxa list and the taxa selection criteria are given in data S2.

### Analysis of postmortem damage patterns

Postmortem damage signatures for metagenomic plant DNA data from Lake Lama core were analyzed with the HOPS (*104*) pipeline (using the function maltextract) and the metaDMG pipeline based on HOLI (*105*) classification tool. Bacterial and fungal DNA damage patterns were investigated using PyDamage v0.72 (*106*), which uses the entire short read metagenomic data assembled into larger DNA fragments (contigs) by MEGAHIT v1.2.9. By remapping the short reads to the contigs, pyDamage estimates the postmortem damage of the contigs (Supplementary Text), which were taxonomically classified with Kraken2 nucleotide 0.0. Results of all approaches were investigated according to the C-to-T substitution frequencies of the first 10 positions (5′ end) and the C-to-T substitution frequencies of the first position of the reads in the respective taxa groups and across sample age (Supplementary Text). In addition, the taxonomic composition resulting from the pipelines were compared using unfiltered (damaged and non-damaged reads) and filtered (damaged reads only) datasets, and a Procrustes analysis on all samples ages and taxa (Supplementary Text) was performed.

### Statistical analysis of the dataset

All statistical analyses (*35*) were carried out with the software R, version 4.0.3. Taxonomic datasets were filtered for taxa occurring at least in three samples. For bacterial dataset, the taxa must occur with a minimum sample read count of 20. Then, we resampled 100 times each taxonomic subset to the basecount of the sample mean value to balance uneven read counts between the samples. The resampling of the fungi had a mean count of 285, the plant data of 3855.5, and the bacteria of 26,627. We followed the github script of Kruse (2019, https://github.com/StefanKruse/R_Rarefaction). All the following statistical analyses were performed on the resampled datasets. For analysis of the long-term soil development and its driving forces, we assessed the time (i.e., the age of the sediment) and the impact of multiple environmental variables (vegetation and temperature). We defined “soil development” as the bacterial communities on one side and fungal soil communities on the other side (whole subsets). Constrained ordination analyses were run to statistically relate the environmental variables to the variation in the composition of either soil community. To yield the variable “vegetation,” we performed a redundancy analysis on the double-square rooted vegetation subset and determined the significant principle component (PC) axes using PCAsignificance(), which provided the first two PC axes were significant. We used the scores of the PC axes 1 and 2 and merged them as a dataframe. We used a reconstruction of the temperature variation in the Northern Hemisphere as further input for the environmental variables to yield the variable “temperature.” The reconstruction of the temperature variation followed the script of Kruse (https://github.com/StefanKruse/R_PastElevationChange). In brief, it is based on the mean temperature reconstructions by Shakun *et al.* (*107*) and Marcott *et al.* (*108*). For the variable “time,” we used the respective ages of the samples as the input.

The K/Ti element ratio data derived from the XRF scanning were used as a proxy for weathering (*67*). The analysis of the XRF data was done by smoothing the scanning data with the function predict (package: stats). All data were plotted with ggplot2 (package: tidyverse).

We analyzed co-occurrence patterns between mycorrhizal fungi and respective tree taxa. To do so, we analyzed the Spearman correlation between the fungal dataset and the plant dataset using the function cor (package: stats). The correlation matrix was converted in a dataframe, and only positively correlated mycorrhizal taxa with a correlation value of at least 0.2 were selected. With the final taxa selection, we plotted the reduced data using the function corrplot (package: corrplot).

For assessing the pH preferences of all data subsets, we assigned the taxa to five categories of preference, namely, acidic, slightly acidic, neutral, slightly alkaline, and alkaline (data S2). We merged the percentages of the slightly alkaline– and alkaline-preferring taxa to yield the overall alkaline preference for plotting. The displayed acidic-preferring taxa are only those being assigned to strong acidic preference.

## Supplementary Materials

### The PDF file includes:

Supplementary Text Figs. S1 to S12 Tables S1 to S5 Legends for data S1 to S3 References
