# Supplementary material for: Postglacial bioweathering, soil nutrient cycling, and podzolization from palaeometagenomics of plants, fungi, and bacteria
Source: Sci Adv. 2025 May 7;11(19):eadj5527. doi: 10.1126/sciadv.adj5527 (PMC12057668; doi:10.1126/sciadv.adj5527)
Supplement: Supplementary file 1 — Supplementary Text Figs. S1 to S12 Tables S1 to S5 Legends for data S1 to S3 References [file sciadv.adj5527_sm.pdf]

Science Advances
AAAS

Supplementary Materials for

Postglacial bioweathering, soil nutrient cycling, and podzolization from palaeometagenomics of plants, fungi, and bacteria

Barbara von Hippel *et al.*

Corresponding author: Ulrike Herzschuh, ulrike.herzschuh@awi.de

Sci. Adv. **11**, eadj5527 (2025)
DOI: 10.1126/sciadv.adj5527

The PDF file includes:

Supplementary Text
Figs. S1 to S12
Tables S1 to S5
Legends for data S1 to S3
References

Other Supplementary Material for this manuscript includes the following:

Data S1 to S3

Taxonomic classification using Kraken, HOPS and HOLI pipeline

The results of the taxonomic classification of bacteria, fungi and plants with Kraken2 at confidence threshold 0.8 (against the nt database) were confirmed by other classifying tools. We applied the HOPS (Heuristic Operations for Pathogen Screening, (104)) pipeline that uses the malt function for classification against the nt database with an identity level of 95% and the HOLI (105) pipeline that uses end-to-end alignments with bowtie2 (version 2.5.1) against a customized database (Data S3). A maximum of 1000 valid and unique alignments (-k 1000) were identified, then sorted and hereafter classified using ngsLCA (v. 1.0.5) with a minimal identity of 95%. For the HOLI pipeline a large customized taxonomic reference database was established, which included nt, RefSeq, Phylonorway, ancient mammals and selected plant genomes (Data S3). Both pipelines use the quality filtered merged reads, whereas the Kraken2 pipeline also uses the paired read fraction. Results from the three pipelines were filtered for the given taxa lists (Data S2). Then, community composition in the three taxa groups (bacteria, fungi and plants) were compared using a procrustes analysis (function procrustes in vegan package) comparing a pair of principal component analysis (PCA) ordinations for the community composition of the sample ages and the taxa (table S1, S2). Prior to PCA analysis, the datasets were filtered for taxa that occur at least three times and have a relative abundance of 0.5%. The Protest test (function protest in vegan package) was used to test the non-randomness (significance) between two ordination configurations. Besides the procrustes and related PROTEST, we further tested the similarity of distances between all samples by comparing the pairwise distances between the three classification approaches Kraken2, HOLI and HOPS by running a combined, instead of a separate, ordination between the pairs of classification approaches. All comparisons using the Mantel test are significant on p.001 level across all three taxonomic groups (bacteria, fungi and plants). In particular the comparison between Kraken and HOLI shows a very high correlation (0.92-0.96) (see Table S3), supporting the consistency between the classification approaches.

Further, we plotted the relative abundance of the major genera of the three taxa groups in bubble charts, supporting the similarity of temporal community compositional change between all three different classification methods (figs. S6). In addition to the analyses with other classifying pipelines, Kraken2 was run on lower confidence thresholds (0.5 and 0.2) and results for the temporal trend of the main genera were compared to Kraken2 0.8 results (fig. S5).

Because Kraken2 and HOLI approaches apply a different classifier and use different reference databases, we compared the compositional signal of both approaches. We found a very high portion of reads, when filtering for taxa used from the Kraken output (table S4) i.e. it is 89.4% of all HOLI reads for Bacteria, 99.4% of all HOLI reads for plants and 87.9% of reads from Fungi (phylum level) and 95.8% of reads from Fungi (genus level) that are covered by the Kraken2 taxa.

In particular, for the plant communities, our comparison resulted in only 1.6% of considered plant reads that are not covered when subtracting a kraken2 plant genus list from the HOLI dataset. Among the remaining reads not covered, we identified two plant taxa *Minuartia stricta* (synonym *Sabulina stricta*) and *Arctagrostis latifolia*, which were assigned to almost all the reads which were not covered by the Kraken2 plant taxa list. Conclusively, this means that a very large proportion of the reads classified by HOLI are also covered by our Kraken2 despite the differences of the classification

approaches. This means that the compositional changes inferred from the Kraken2 data would be very similar to that of the HOLI approach.

Ancient origin of bacterial, fungal and plant reads: methods, results and discussion

Conclusion: Based on the results from post mortem damage pattern analyses of bacterial, fungal and plant reads, we conclude that the temporal compositional pattern of plants, fungi, and bacteria which we interpret with respect to soil development in the manuscript are derived from ancient communities i.e. corresponding to the dating of the sediments. For all three groups we show the characteristic damage pattern.

It is inevitable to prove the ancient origin of metagenomics derived DNA reads to differentiate ancient reads from modern contaminants. On top, it is necessary to ensure that ancient organisms did not continuously survive and replicate in the sediment, biasing the data. A multitude of bioinformatic tools has been established recently. Amongst those, there is the HOPS pipeline (104), mapdamage (109) and PyDamage (106). The assessment of damage patterns for ancient DNA reads has so far been well established for plants (e.g. (110, 111)) and mammals (112). However, microorganisms including bacteria and fungi have scarcely been studied (115).

Methods Bacteria and Fungi: We implemented damage analyses using PyDamage which is considered the most appropriate bioinformatic tool to assess the ancient origin of microorganisms. This pipeline analyzes contigs (i.e. de novo assembled larger sequences from short read shotgun sequencing data including classified and unclassified reads) instead of single short and taxonomically classified reads like it is done in other post mortem damage tools, like MapDamage or HOPS. Including classified and unclassified reads, PyDamage keeps a much higher share of reads in the analyses of post mortem damage patterns and such can also assess ancient origin of taxa which are poorly reflected in databases and/or occurring with few reads. We employed the PyDamage v0.72 (106) to analyze damage patterns from bacterial and fungal DNA. The short read metagenomic data (quality checked merged and paired reads) went through an error-correction method using BBtools v38.87 (113) and were subsequently assembled into contigs using MEGAHIT v1.2.9 (114) with deviations from default mode including: *-minimum contig length: 300 and preset: meta-large* and with the default k-mer. Contigs derived from de novo assembly were aligned to the assembled contigs using BWA-MEM v0.7.17 with default parameters, followed by data conversion with Samtools v1.16.1 and BamTools 2.5.2. PyDamage was then run with the analyze function, taking the final alignment output (BAM file) as input to estimate the damage of the contigs. The contigs were then taxonomically classified using Kraken2 against the nt database (downloaded in October 2022) with a confidence threshold 0.0 (default mode). Subsequently, the outputs from PyDamage (damage estimates for the contigs) and Kraken2 (taxonomic assignment of contigs) were merged by contig_id. Contigs were then filtered for bacteria and fungi (Data S2). A prediction accuracy of >=0.6 and a contig length >=1000bp was used to filter for the data for ancient contigs. C-to-T frequency for the first 10 positions of the 5'end was plotted per each taxa group.

Results and discussion for bacteria and fungi: The PyDamage results of the bacteria and fungi investigated in our study clearly prove that a substantial fraction of damaged reads (for Bacteria 80% of the reads and for Fungi 56% of the reads, which is comparable to previous studies, like Kjaer et al. (29) (damaged fraction of Archaea reads of about 84 %). The damage fraction in our data shows the

characteristic ancient pattern, which is the increased C-to-T substitution rate (Supplementary Fig. 8A & 9A) confirming the necessary requirement to consider a community of ancient origin. Furthermore, we can show that the damage of the reads, exemplified by the share of C-to-T substitution rate at the first position, increases with age (supplementary Fig. 8B & 9B, Pearson correlation and p-value for Bacteria: $p<0.001$ and, Fungi: $p<0.001$) confirming the sufficient requirement to consider a community of ancient origin. (This analysis was restricted to samples from the Holocene because of the strong change in the sediments and microbial community.) Exemplarily, we also show the mean C-to-T frequency for the first 10 positions of the 5'end for selected taxa (Bacteria: *Bradyrhizobium*; Fungi: *Rhizophagus irregularis*). The increase of C-to-T frequency in the first read position across sample ages provides additional evidence for the ancientness of the DNA molecules of bacteria and fungi.

We also confirm using procrustes analysis that the composition of the community showing ancient pattern has a significantly similar compositional change pattern as the community based on all reads (non-damaged and damaged) (Supplementary Table S5. i.e. see for Bacteria and Fungi targeting sites and taxa of the community datasets, protest $p < 0001$). This rejects the idea of substantial post-sedimentation impact on microbial community and also indicates that all reads (i.e. not only those showing ancient pattern) can be used in downstream analyses. We also confirm that the compositional pattern of the community investigated by the PyDamage pipeline is significantly similar to the compositional pattern of the Kraken2 pipeline (confidence 0.8) community (Supplementary Table. S5, Bacteria $r=0.5$, $p<0.001$ and Fungi $r=0.4$, $p=0.014$) investigated for soil development in this study.

Discussion: For fungi, there is only one study assessing DNA damage, using DNA derived from Oetzi gut (115). Compared to DNA derived from lake sediment, the fungal DNA in Oetzi gut is likely to be better preserved due to permanent freezing. The splice-site combinations of fungi and plant DNA are differing (116). While some plant species include up to 90 % of repetitive sequences in their genome (119), asco- and basidiomycetes usually have less than 5 % repetitive DNA (118). These repetitive sequences are susceptible to DNA damage (120), indicating their potential for strong damage patterns. This suggests that the improvement and adaptation of yet existing damage pattern analysis tools is important when assessing damage of genomes from non-plant kingdoms, as differently constituted genomes will probably degrade in a different way. On top, fungal databases are known to be lacking species (120), pointing out the complexity to find appropriate reference genomes. A previous study on fungal ancient DNA metabarcoding revealed a drastic increase in sequenced fungal genomes during the last 10 years (121), suggesting that a deeper sequencing of fungal genomes will facilitate the assessment of ancient patterns. A review by Capo et al. (122) pointed out the problem of differentiating between dormant but still-alive and dead ancient bacteria. Our study suggests that the majority of reads are of ancient origin. However, variations in the standard protocol such as the adaptation of the extraction protocols to short reads or the independent extraction of intra- and extracellular DNA as suggested by Capo et al. (122) may be used to prove this result.

Methods plants: Post mortem damage signatures for metagenomic plant DNA data from Lake Lama core were analysed with the HOPS (104) pipeline (using the function maltextract) and the metaDMG pipeline (using the metaDMG toolkit). The HOPS pipeline uses the malt alignment against the nt database and the maltextract function that distinguishes taxonomically classified reads into *default* (all taxonomic classified reads) and *ancient* (a subset of all reads that have at least one mismatch in

the first 5 positions (5' end)). We applied the HOPS pipeline on the quality filtered short read metagenomic data (merged reads only) and used maltextract for a selection of taxa (Data S2). The C-to-T substitution frequencies of the first 10 positions (5' end) of the as *default* classified reads are plotted for all plant taxa and exemplarily for the genus *Salix*. The relationship between the C-to-T substitution frequencies of the first position in the reads across sample age was investigated. The metaDMG toolkit allows an automated ancient pattern analysis on the results obtained from the HOLI pipeline (105). We extracted the metaDMG results for selected plant taxa based on the taxa list (Data S2) and investigated the C-to-T substitution frequencies of the first 10 positions for all sample ages and the C-to-T substitution frequencies of the first position in the taxa across the sample ages. Relationships between C-to-T substitution frequencies of the first position and age were limited to sample ages between 0 and 15,000 years, as this time period is characterized by a stable depositional mode in Lake Lama. For plots showing the C-to-T substitution frequencies at the first 10 positions a mean value and the standard deviation was included in the plot, whereas for the C-to-T substitution frequency plots of the first read position against age a linear regression was (lm function in R) used to predict the C-to-T substitution frequency of the first read position on the sample age.

Results plants: Our inference about the ancient origin of bacteria and fungi are also in line with our new results gained from a comprehensive damage pattern analysis of plant reads using two different bioinformatic pipelines HOPS and HOLI_metaDMG (Supplementary text "Taxonomic classification using Kraken, HOPS and HOLI pipeline" and "Result and discussion ancient origin of plant, fungi and bacteria communities"). We present the results of all plant reads and, exemplarily, the results for *Salix* i.e. the taxon with the highest read numbers. The results of the two pipelines revealed the ancient damage pattern i.e. the increased C-to-T substitution rate towards the end of the reads (supplementary Fig. S7). As further confirmation of the ancient origin of the plant reads, the results of all pipelines indicate an increase of C-to-T substitution of the first position with age (supplementary Fig. S3 & S4, Pearson p-value: <0.001). Using procrustes analyses we also proved that the taxonomic composition and compositional pattern of the plant communities of all pipelines are similar (supplementary table S2, protest p values all <0.001).

Do the plants, fungi and bacteria show differences in the ancient pattern?

To answer this question, we also applied pyDamage to plant reads. The results of all reads of the different taxonomic groups yielded an average of C-to-T change in the first position of 0.22 for plants, 0.16 for bacteria and 0.18 for fungi. The proportion of reads to ancient contigs (using a prediction accuracy > 0.6) showed that 59.7% of plant reads (1,557,792 reads), 79.3% of bacterial reads (399,002,412 reads) and 55.5% fungal reads (5,918,560 reads) were assigned to contigs indicated as damaged. However, using pyDamage for plant damage patterns seems to be problematic compared particularly to Bacteria, because plants have much less reads/contigs in the total dataset, in particular during the late Holocene periods (fig. S9). Therefore, we did not further investigate this dataset. However, based on these results we can clearly show damage patterns for a substantial proportion of reads for all three taxonomic groups, but we have no clear picture of taxon-specific damage patterns, which was not the focus of this study.

Argumentation that patterns derive from palaeo communities of fungi and bacteria not contamination

Here, we provide arguments why we consider our observed and interpreted pattern to derive from palaeo communities of fungi and bacteria not mainly from biota growing during post-sediment coring handling or originating from contamination.

1) The conditions during storage in the fridge represent the conditions at the lake bottom with temperatures of about 4 degrees.

2) A study by Seeber et al. (121) including the PG1341 core from Lake Lama (used for this study) assessed the impact of time after coring as well as the long-term storage on the community composition of fungi and revealed no indication of the long-term storage on the communities.

3) The subsampling itself was conducted under clean conditions, using clean knives and scalpels for each sample, while only subsampling the inner parts of the core. An in-detailed description of the sampling process is described in the Methods section of the manuscript.

4) Our record shows a clear temporal variability over long time scales, while nearby samples revealed similar compositions. This would not be expected from pure contamination.

5) The recovered temporal trends and patterns for fungi, bacteria, and plants are overall similar including distinct shifts from the Late Glacial to the Holocene.

6) The protocol for DNA extraction and library preparation is optimized to target short DNA fragments from DNA which is attached to minerals, but not from larger fragments from living cells.

Taken these arguments together, we are confident that the compositional pattern that we interpret in this study originates mainly from an original signal at the time of sediment deposition not from signal introduced after sediment coring.

Supplementary Figures

Fig. S1

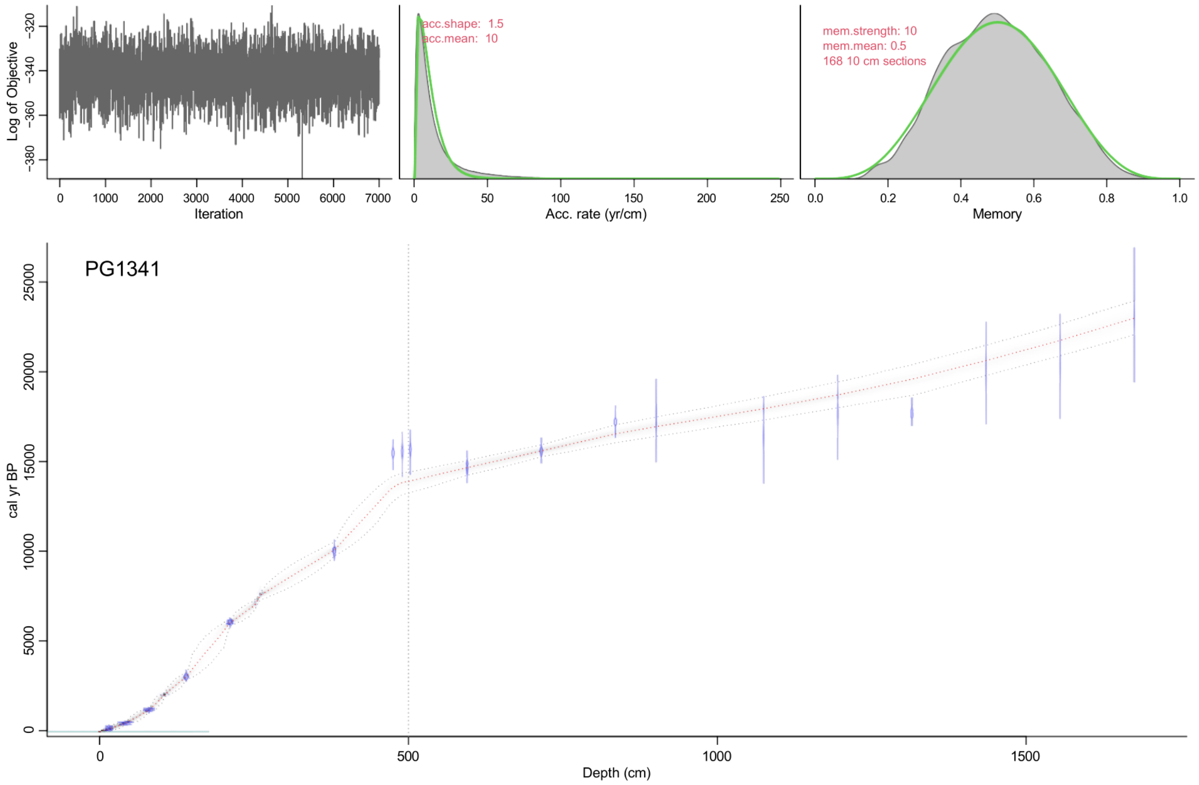

Fig. S1: Refined age-depth model of Lake Lama, core PG1341.

We re-evaluated the age-depth model of the core PG1341, which was previously published in von Hippel et al. (36). A better correlation of the overlaps using the data for the magnetic susceptibility as well as using the TOC data of Andreev et al. (37) as comparison to the parallel core yielded a further overlap of 145 cm between the core segments 5 and 6. Therefore, the core is 1.45 m shorter than initially thought. The new age-depth model is publicly available under https://doi.pangaea.de/10.1594/PANGAEA.963262.

Fig. S2

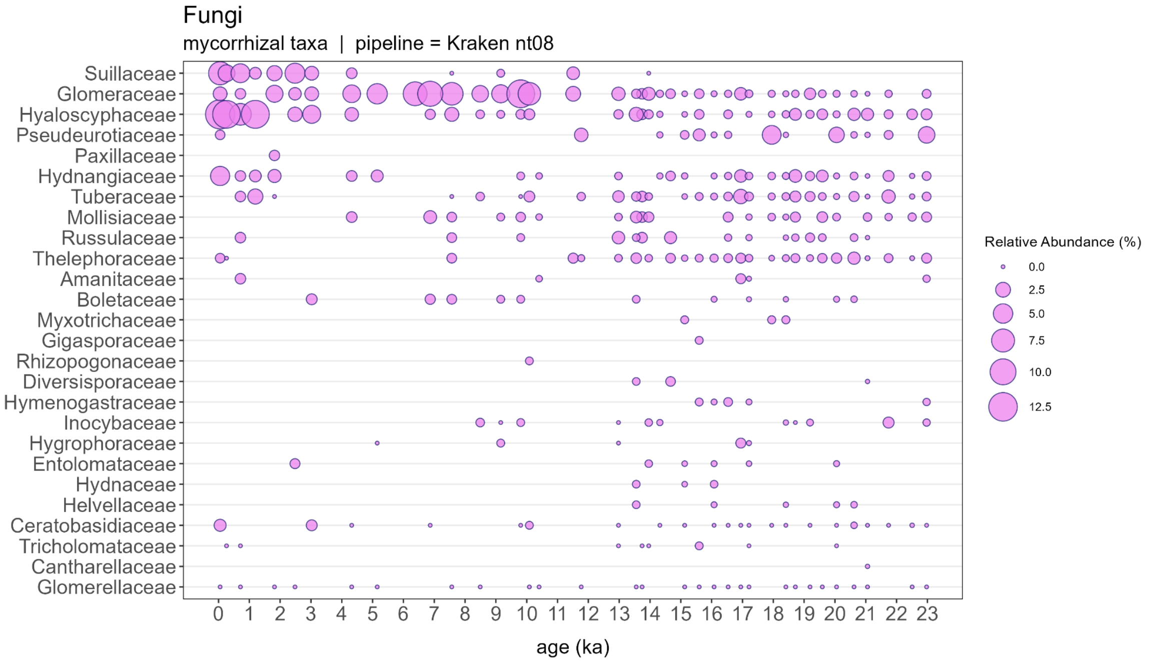

Fig. S2: Mycorrhizal families (1297 read counts totally) taxa recovered from lake Lama sediment and their respective relative abundance (12 % of the total fungal reads) throughout the sediment. The size of the bubbles marks the relative abundance.

Fig. S3

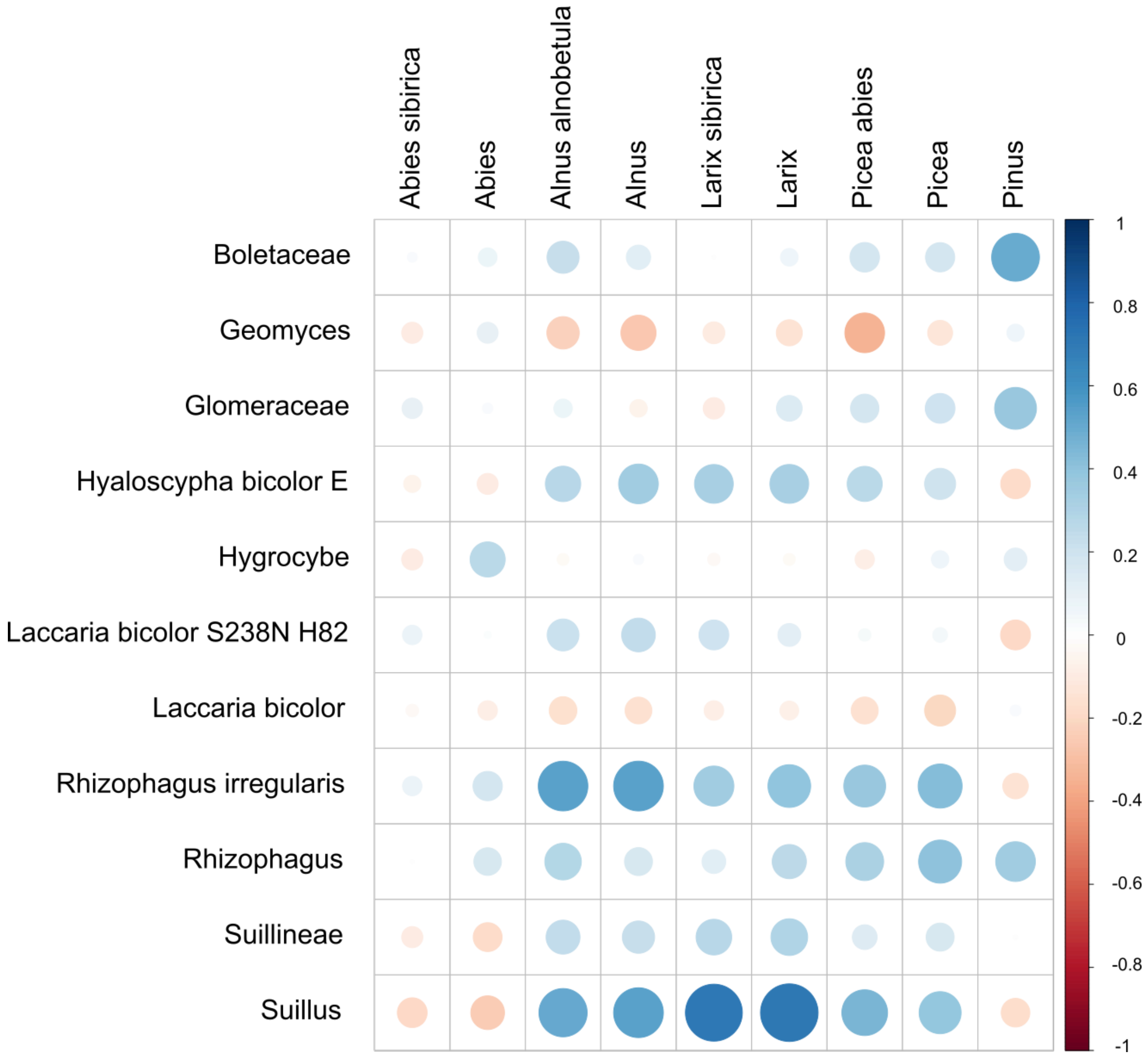

Fig. S3: Co-occurrence analysis between trees and mycorrhizae. Only positively correlated mycorrhizal taxa are displayed. The size and color of the circle represents the degree of correlation.

Fig. S4

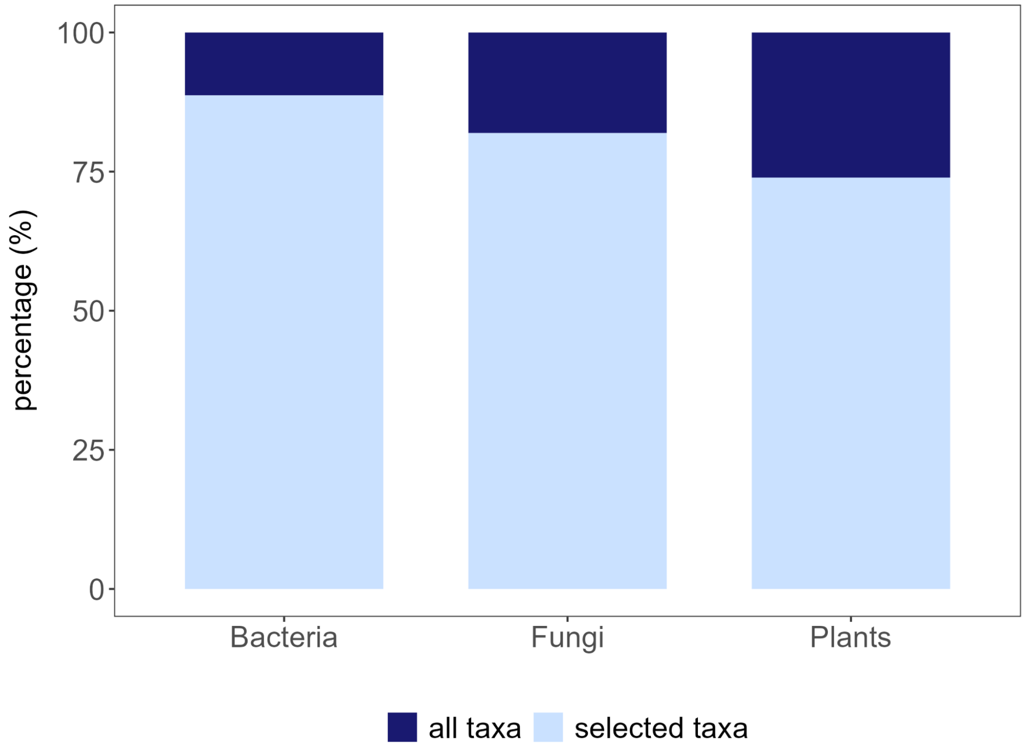

Fig. S4 Proportion of taxonomically classified reads from the total Kraken nt 0.8 dataset (dark blue=100%) and the proportion of reads from selected taxa based on the taxa lists for bacteria, fungal and plant (Data S2).

Fig. S5

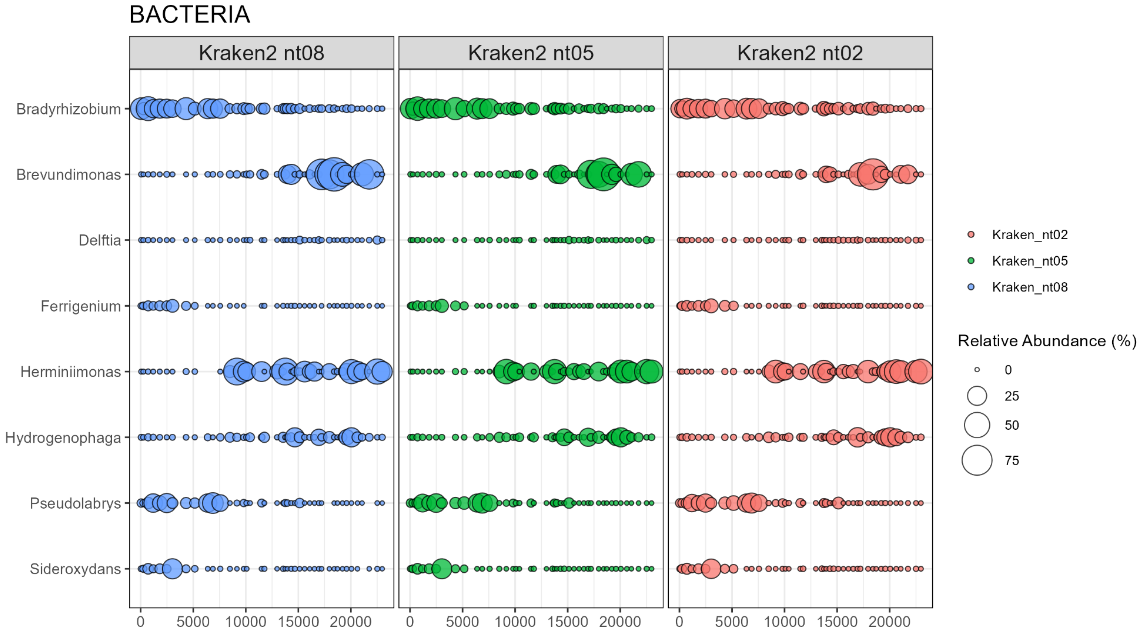
BACTERIA
Kraken2 nt08
Kraken2 nt05
Kraken2 nt02
Bradyrhizobium
Brevundimonas
Delftia
Ferrigenium
Herminiimonas
Hydrogenophaga
Pseudolabrys
Sideroxydans
0
5000
10000
15000
20000
Kraken_nt02
Kraken_nt05
Kraken_nt08
Relative Abundance (%)
0
25
50
75

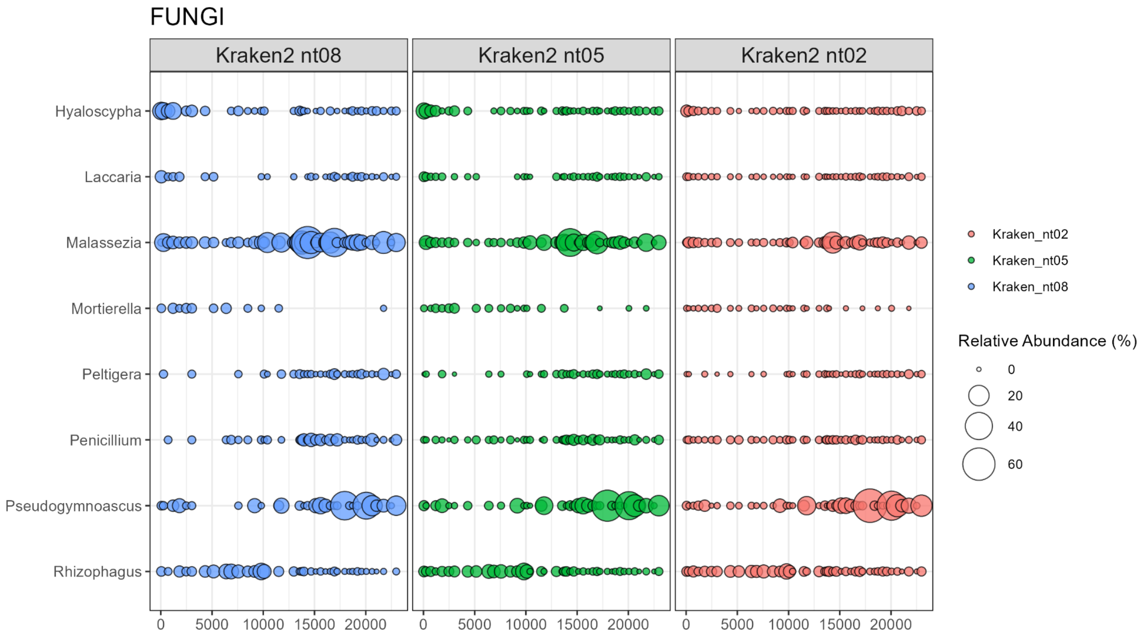
FUNGI
Kraken2 nt08
Kraken2 nt05
Kraken2 nt02
Hyaloscypha
Laccaria
Malassezia
Mortierella
Peltigera
Penicillium
Pseudogymnoascus
Rhizophagus
0
5000
10000
15000
20000
Kraken_nt02
Kraken_nt05
Kraken_nt08
Relative Abundance (%)
0
20
40
60

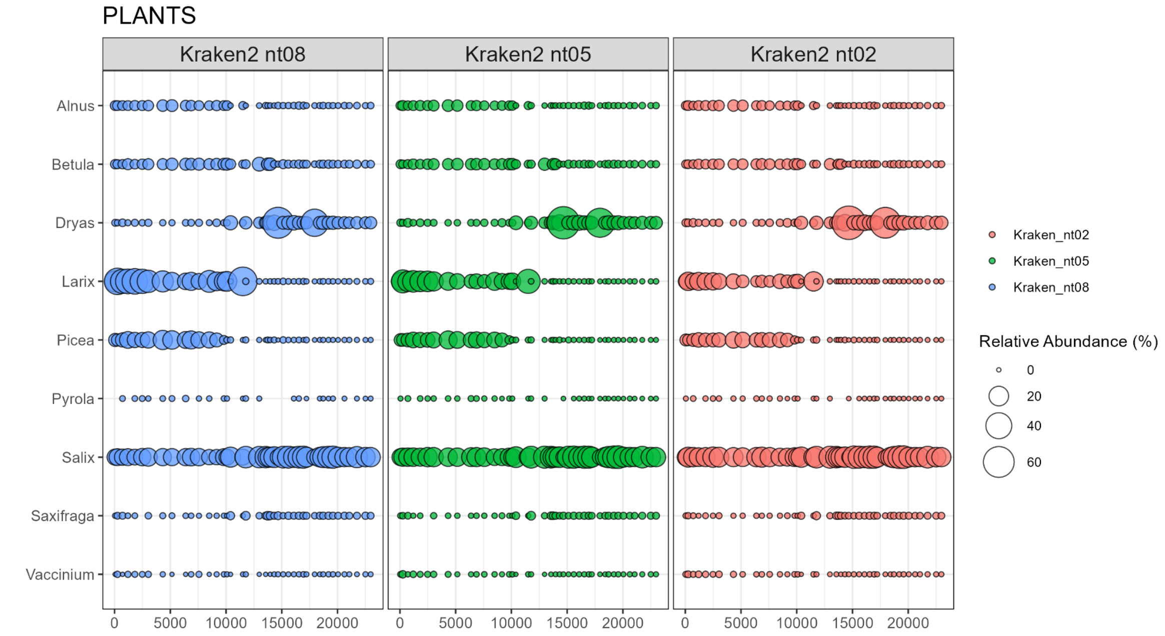

Fig. S5 Comparison of taxonomic temporal trends between the taxonomic composition of major genera in the three taxa groups using Kraken2 with different confidence thresholds (0.8, 0.5. 0.2) against the nt database. **A|** Bacteria (total reads for dominant bacteria genera: nt0.2: 6730806, nt0.5: 3048858 nt0.8:2236217). **B|** Fungi (total reads for dominant fungi genera: nt0.2: 26897, nt0.5: 10174, nt0.8: 5234). **C|** Plants (total reads for dominant plant genera nt0.2: 504153, nt0.5: 224193, nt0.8: 94876)

Fig. S6

A

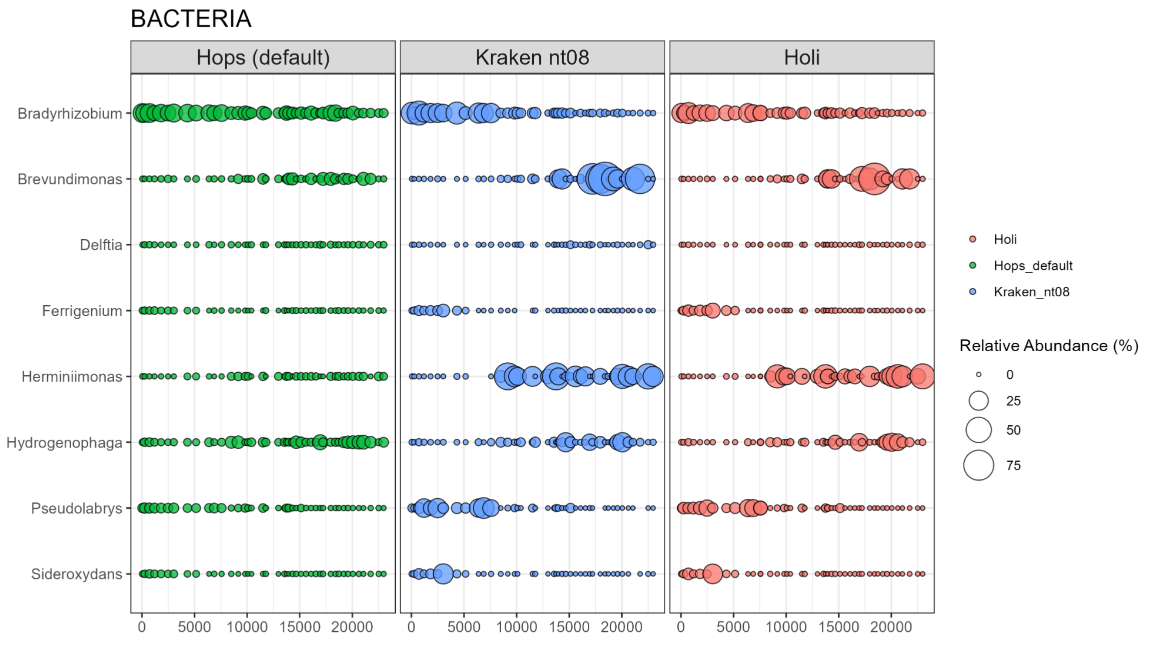
BACTERIA
Hops (default)
Kraken nt08
Holi
Bradyrhizobium
Brevundimonas
Delftia
Ferrigenium
Herminiimonas
Hydrogenophaga
Pseudolabrys
Sideroxydans
0
5000
10000
15000
20000
Holi
Hops_default
Kraken_nt08
Relative Abundance (%)
0
25
50
75

B

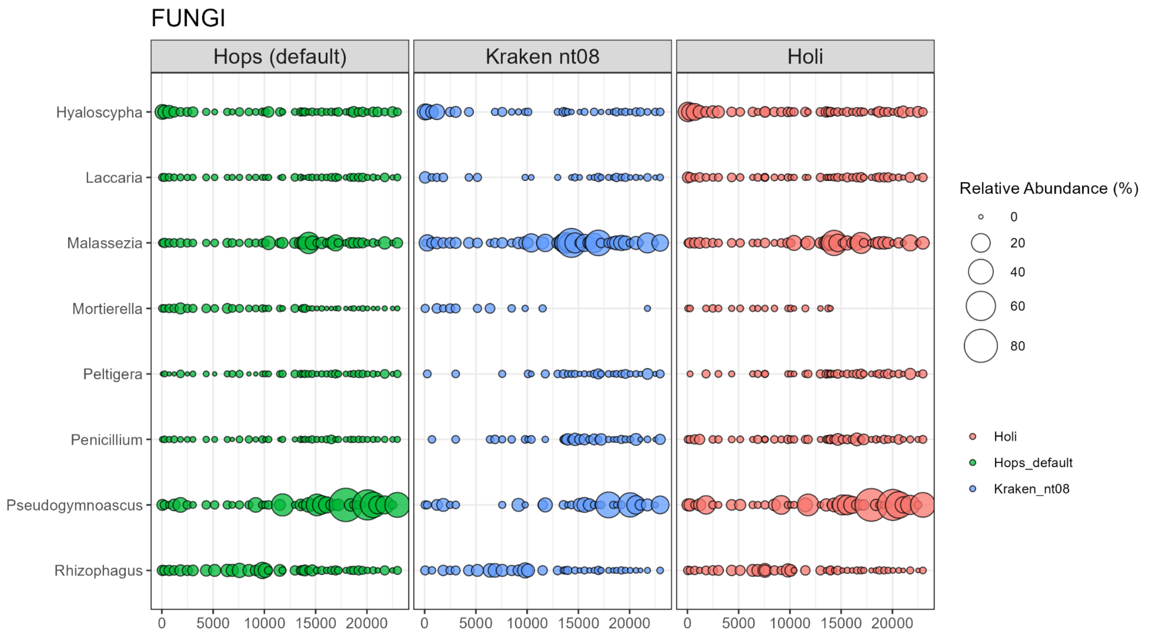
FUNGI
Hops (default)
Kraken nt08
Holi
Hyaloscypha
Laccaria
Malassezia
Mortierella
Peltigera
Penicillium
Pseudogymnoascus
Rhizophagus
0
5000
10000
15000
20000
Relative Abundance (%)
0
20
40
60
80
Holi
Hops_default
Kraken_nt08

C

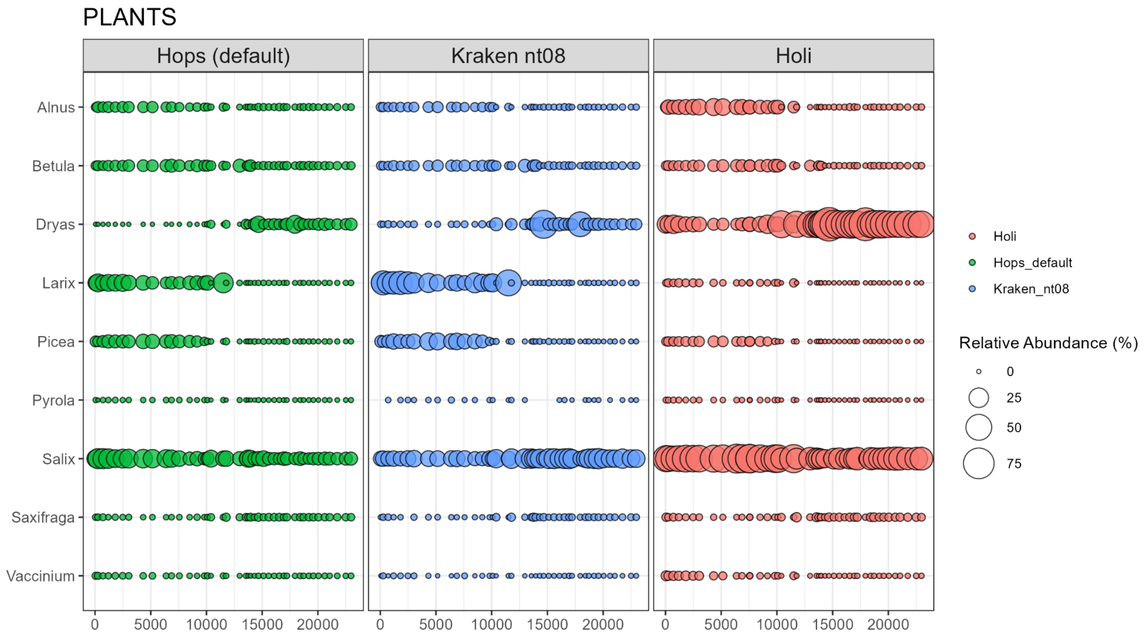

Fig. S6 Comparison of taxonomic temporal trends between the taxonomic composition of major genera in the three taxa groups (A, B, C) using three different classification tools (Kraken2 using k-mer-based classification against the nt database with a confidence threshold of 0.8; HOPS using a malt alignment against the nt database with an identity level of 95%; HOLI using bowtie2 alignments against a customized database (Data S3).

Fig. S7

A

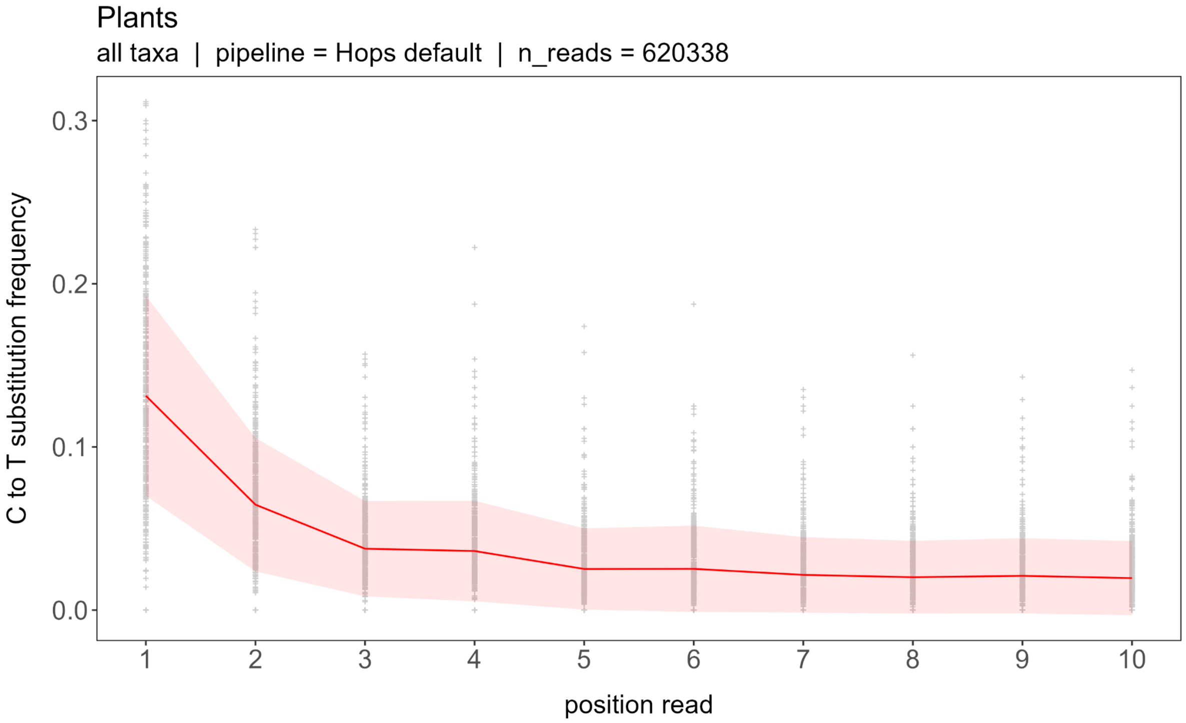
Plants
all taxa | pipeline = Hops default | n_reads = 620338
C to T substitution frequency
0.0
0.1
0.2
0.3
1
2
3
4
5
6
7
8
9
10
position read

B

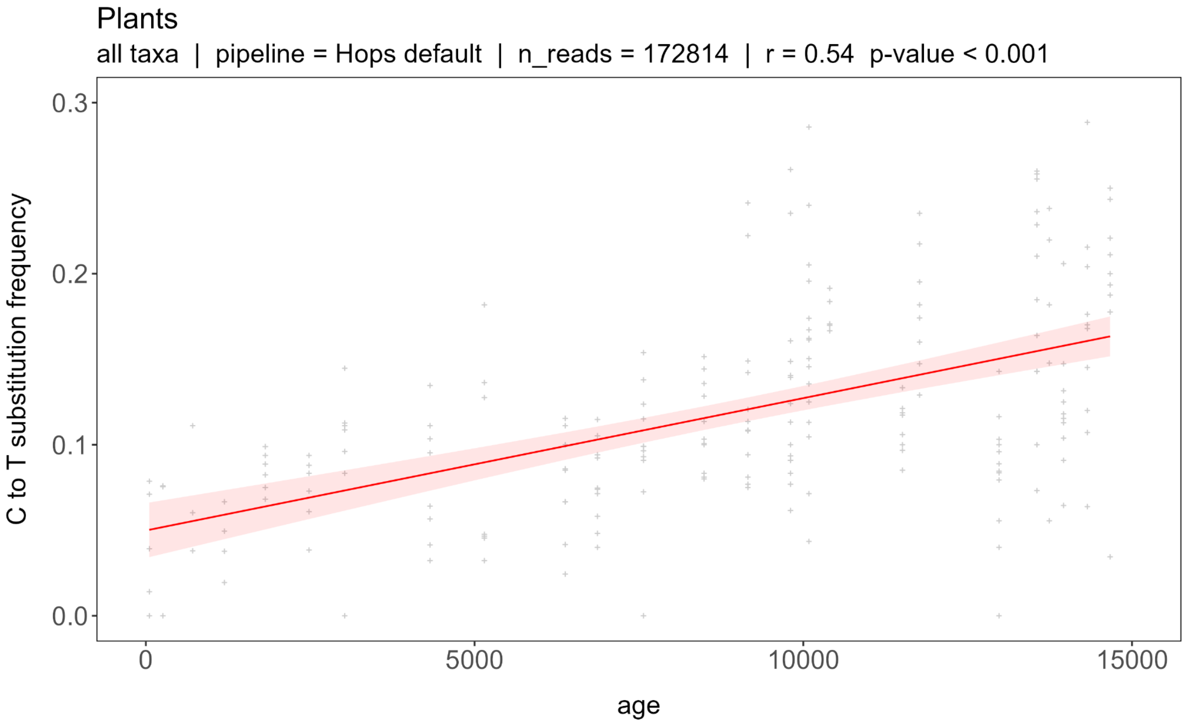
Plants
all taxa | pipeline = Hops default | n_reads = 172814 | r = 0.54 p-value < 0.001
C to T substitution frequency
0.0
0.1
0.2
0.3
0
5000
10000
15000
age

C

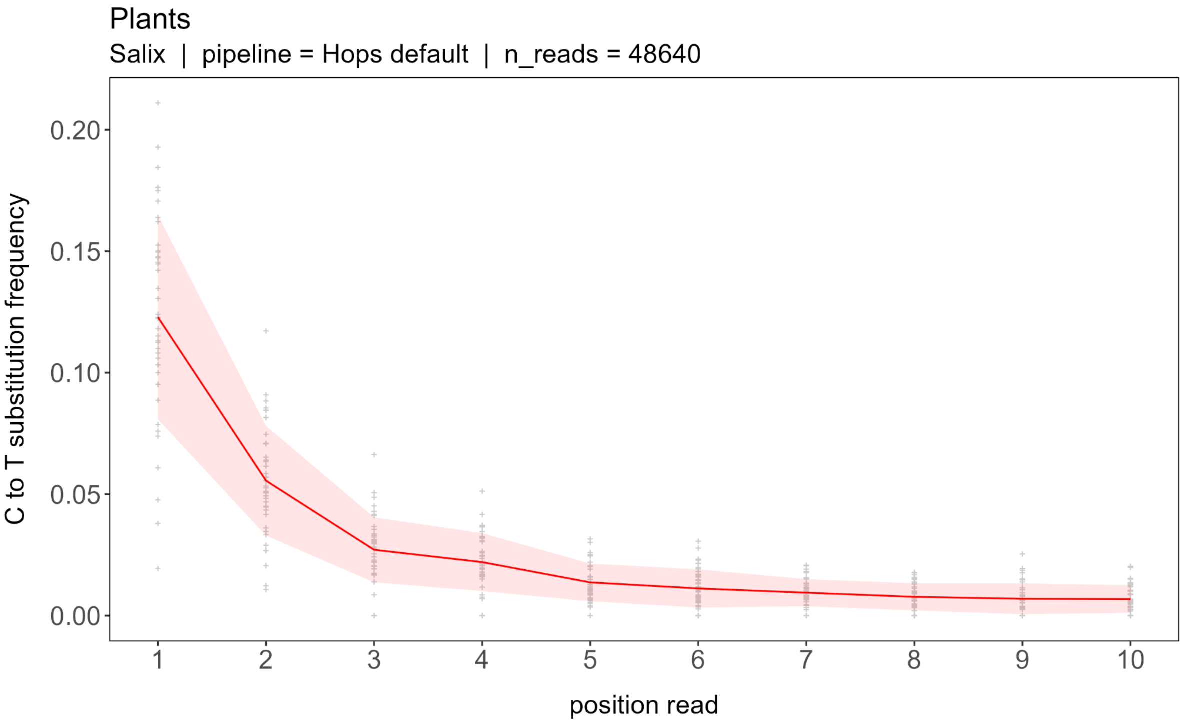

D

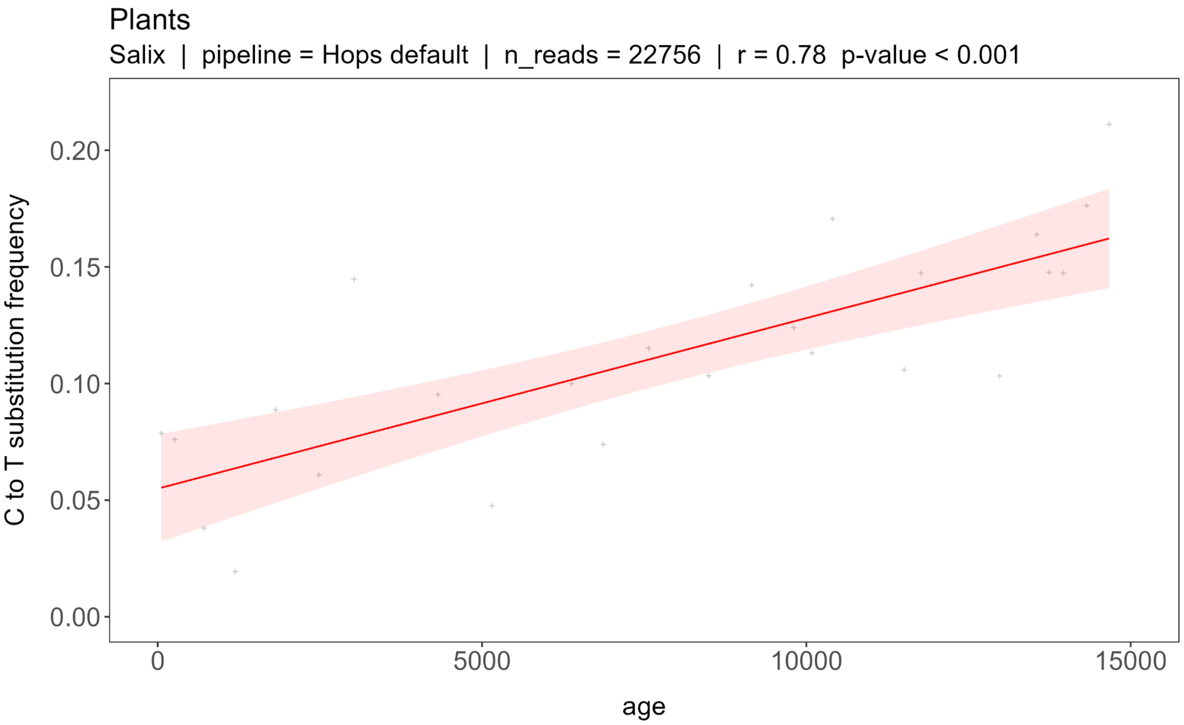

Fig. S7 The frequency of C-to-T substitutions in plants resulted from the HOPS pipeline (default reads). **A|** C-to-T substitution frequencies for the first ten read positions for all plant taxa (>=100 read counts) from all sample ages. **B|** The C-to-T substitution frequencies for all plant taxa (>=100 read counts) at the first position increases significantly with sample age (=<15,000 years). **C|** C-to-T substitution frequencies for the first ten read positions for all *Salix* reads from all sample ages. **D|** The C-to-T

substitution frequencies for all *Salix* reads at the first position increases significantly with sample age (=<15,000 years).

Fig. S8

A

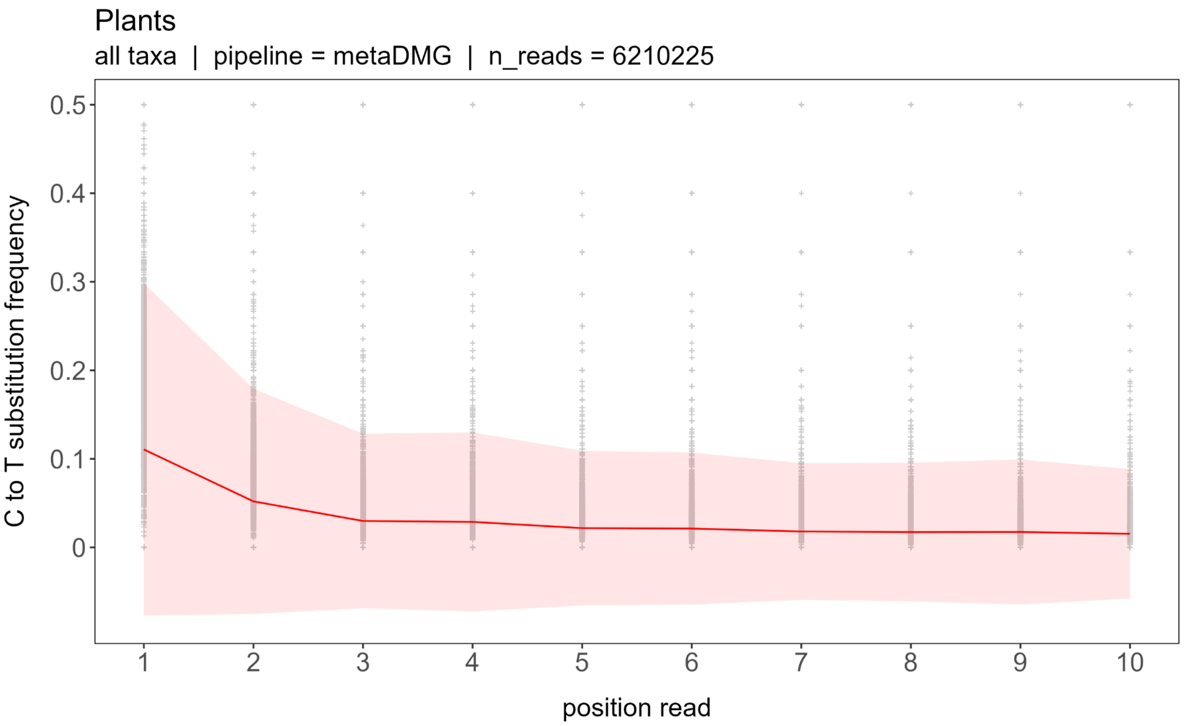
Plants
all taxa | pipeline = metaDMG | n_reads = 6210225
C to T substitution frequency
0.5
0.4
0.3
0.2
0.1
0
1
2
3
4
5
6
7
8
9
10
position read

B

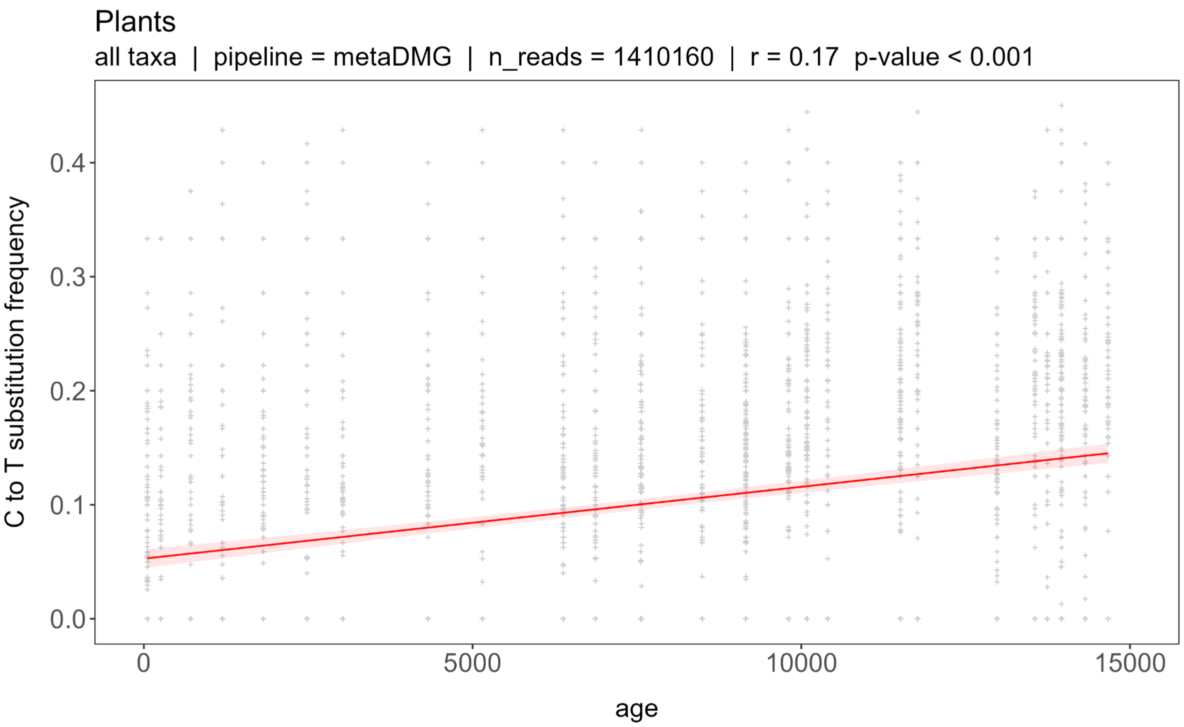
Plants
all taxa | pipeline = metaDMG | n_reads = 1410160 | r = 0.17 p-value < 0.001
C to T substitution frequency
0.4
0.3
0.2
0.1
0.0
0
5000
10000
15000
age

C

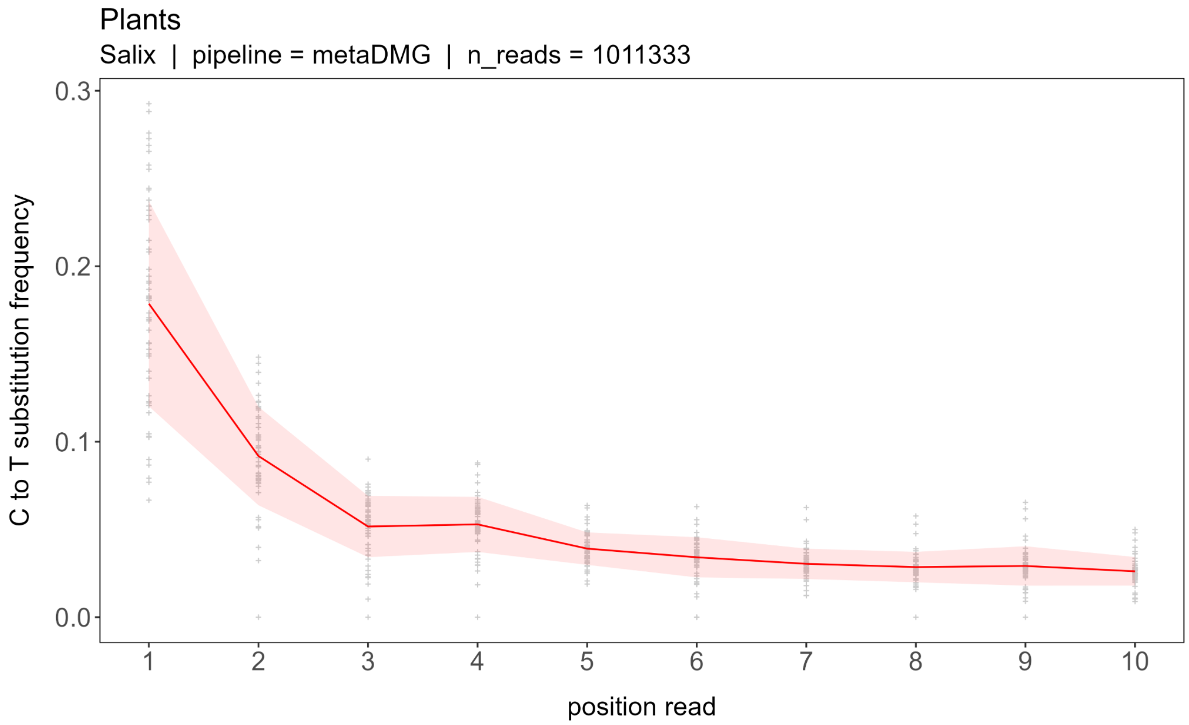

D

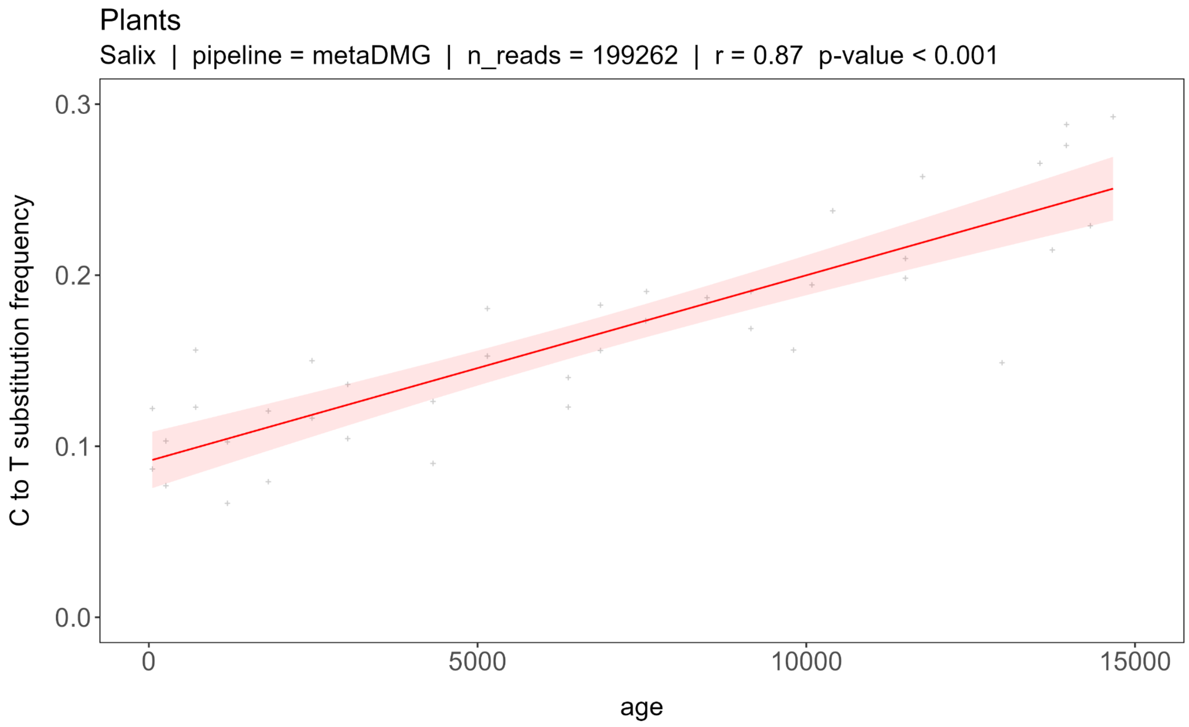

Fig. S8 HOLI-metaDMG results for all plants and *Salix* without any pre-filtering of the data. **A|** C-to-T substitution frequencies for the first ten read positions for all plant reads from all sample ages. **B|** The C-to-T substitution frequencies for all plant reads at the first position increases significantly with sample age (=<15,000 years). Plot was cut at 0.45 (= 5% of the data points). **C|** C-to-T substitution frequencies for the first ten read positions for all *Salix* reads from all sample ages. **D|** The C-to-T

substitution frequencies for all *Salix* reads at the first position increases significantly with sample age (=<15,000 years).

Fig. S9

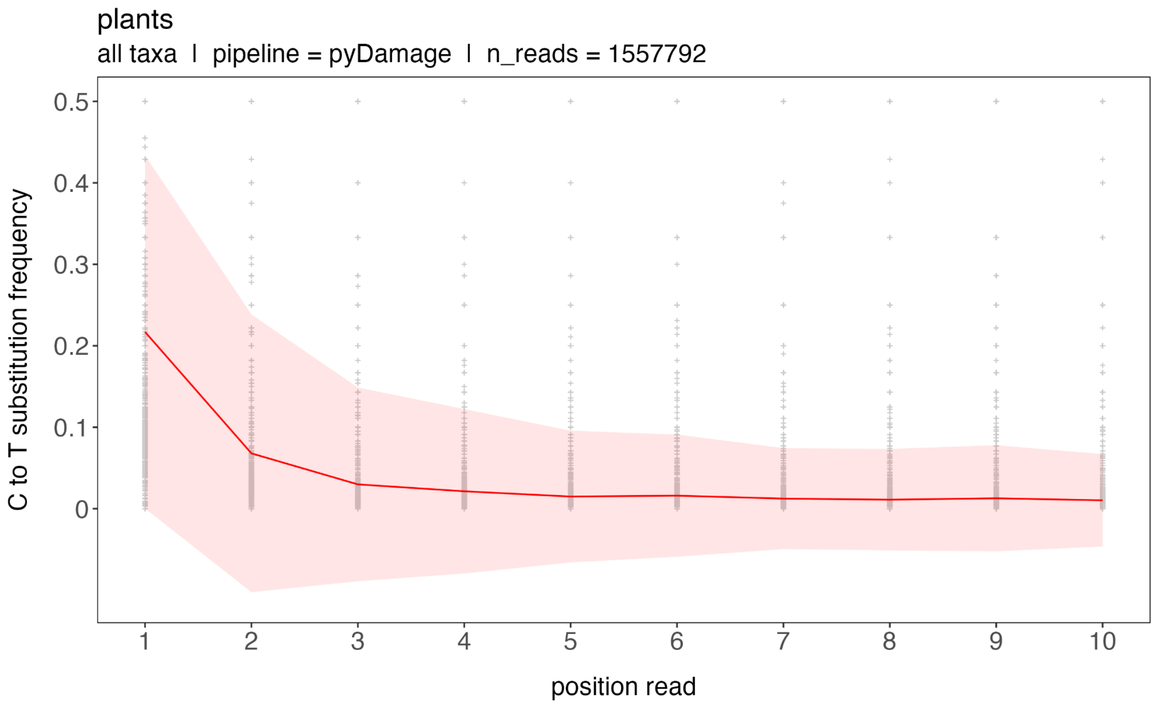

Fig. S9 PyDamage results for Plants. Data was filtered for a prediction accuracy >=0.6 and a contig length >=1000 bp. C-to-T substitution frequencies for the first ten read positions for all plant contigs from all sample ages.

Fig. S10

A

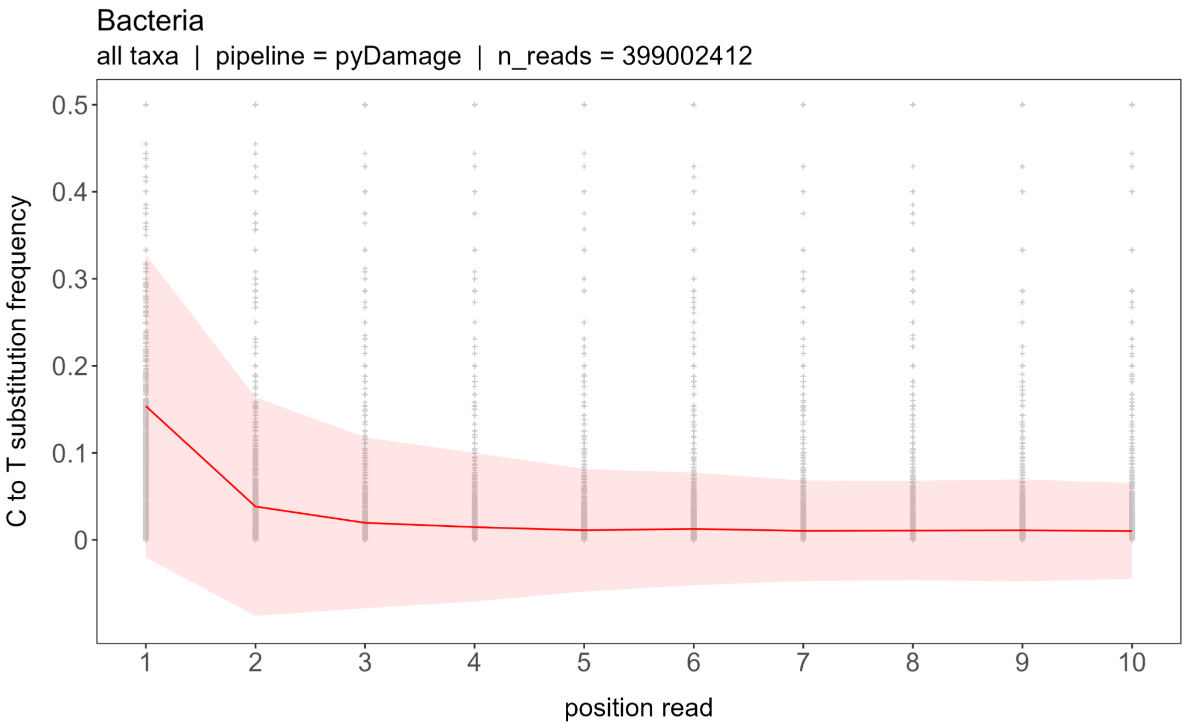

Bacteria
all taxa | pipeline = pyDamage | n_reads = 399002412
C to T substitution frequency
0.5
0.4
0.3
0.2
0.1
0
1
2
3
4
5
6
7
8
9
10
position read

B

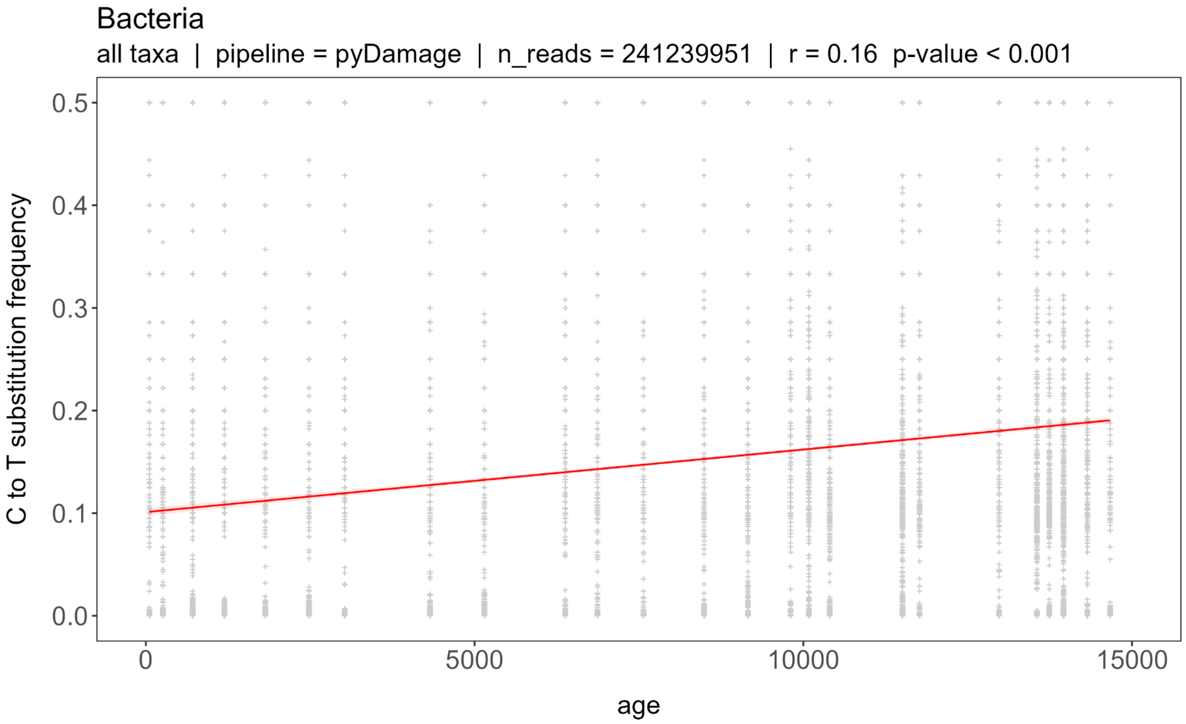

Bacteria
all taxa | pipeline = pyDamage | n_reads = 241239951 | r = 0.16 p-value < 0.001
C to T substitution frequency
0.5
0.4
0.3
0.2
0.1
0.0
0
5000
10000
15000
age

C

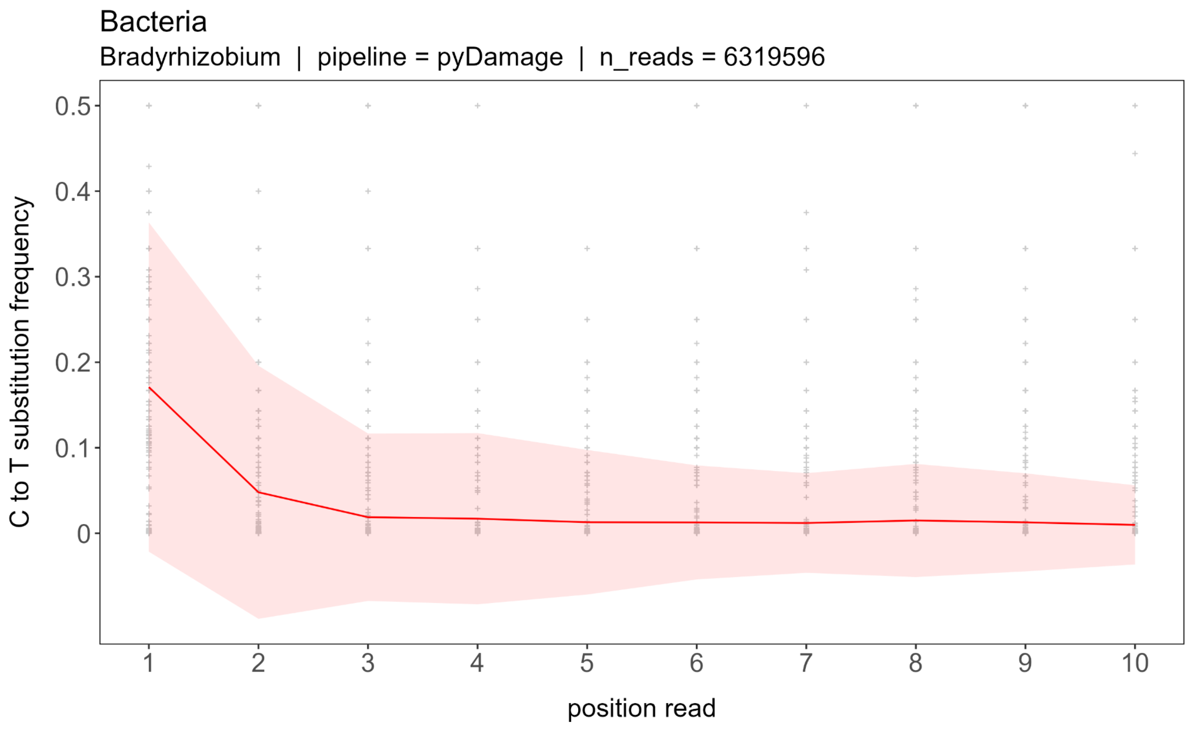

D

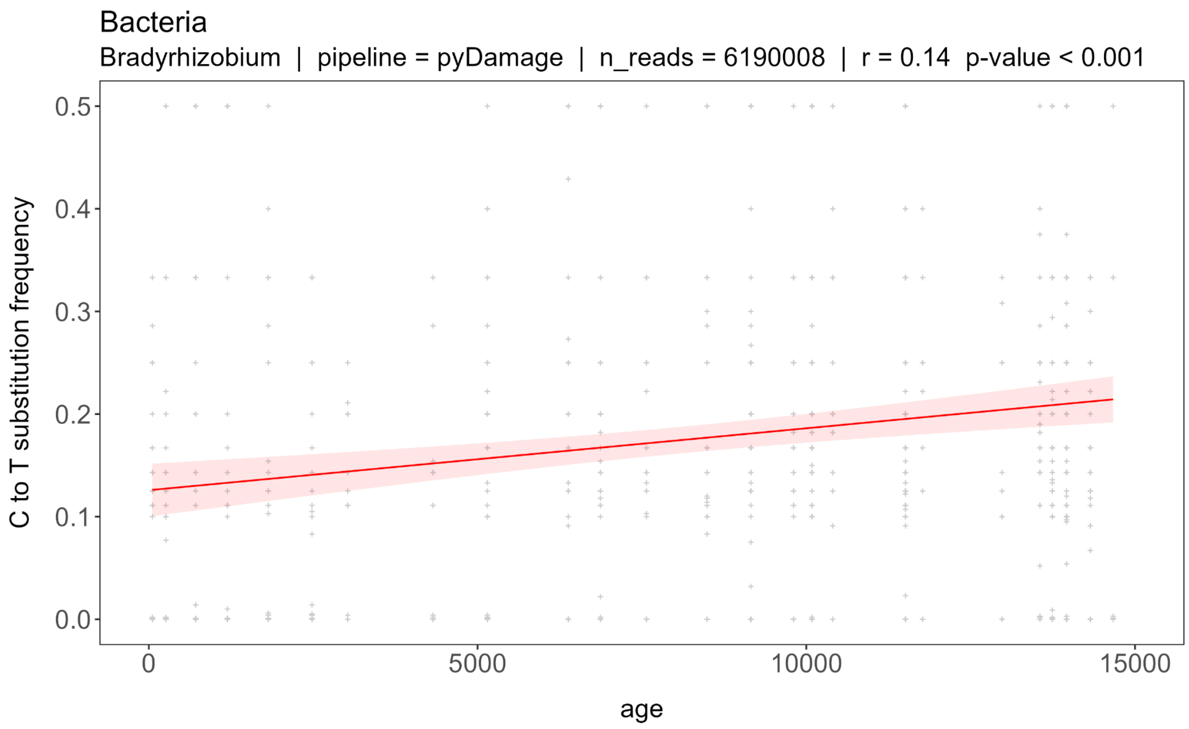

Fig. S10 PyDamage results for Bacteria. Data was filtered for a prediction accuracy >=0.6 and a contig length >=1000 bp. **A|** C-to-T substitution frequencies for the first ten read positions for all bacterial contigs from all sample ages. **B|** The C-to-T substitution frequencies for all bacterial contigs at the first position increases significantly with sample age (=<15,000 years). Plot was cut at 0.5 (= 5% of the data points were removed). **C|** C-to-T substitution frequencies for the first ten read positions for all

Bradyrhizobium contigs from all sample ages. **D|** The C-to-T substitution frequencies for all *Bradyrhizobium* contigs at the first position increases significantly with sample age (=<15,000 years).

Fig. S11

A

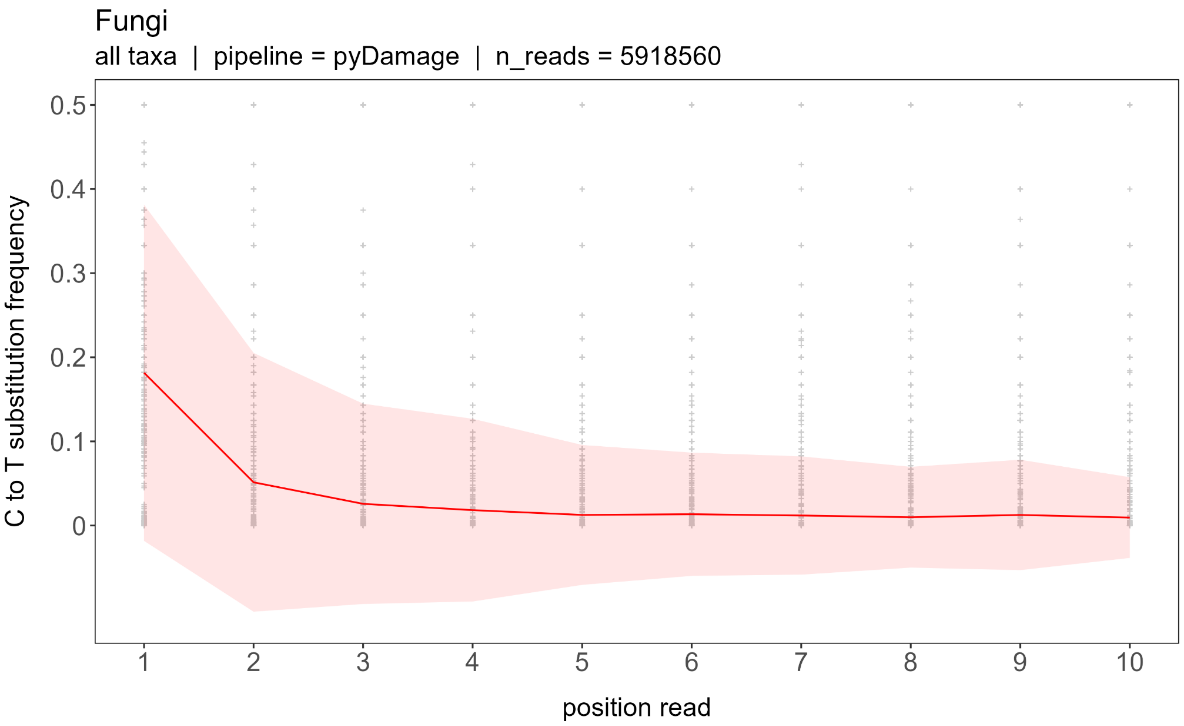
Fungi
all taxa | pipeline = pyDamage | n_reads = 5918560
C to T substitution frequency
0.5
0.4
0.3
0.2
0.1
0
1
2
3
4
5
6
7
8
9
10
position read

B

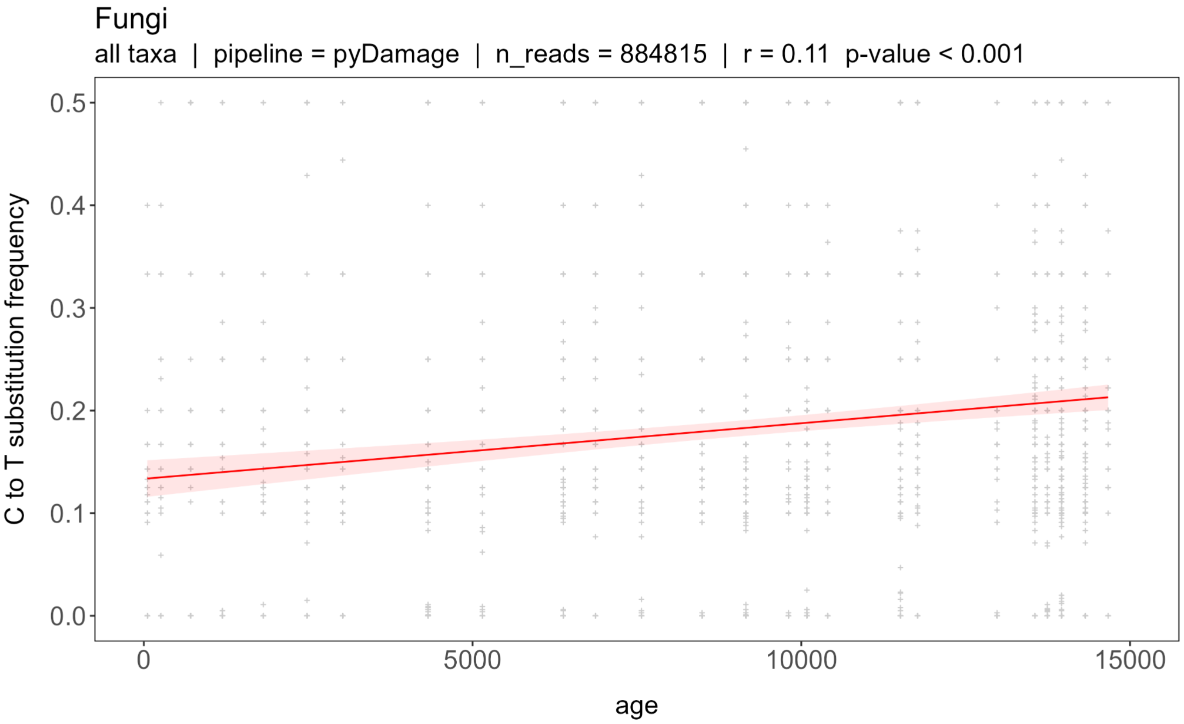
Fungi
all taxa | pipeline = pyDamage | n_reads = 884815 | r = 0.11 p-value < 0.001
C to T substitution frequency
0.5
0.4
0.3
0.2
0.1
0.0
0
5000
10000
15000
age

C

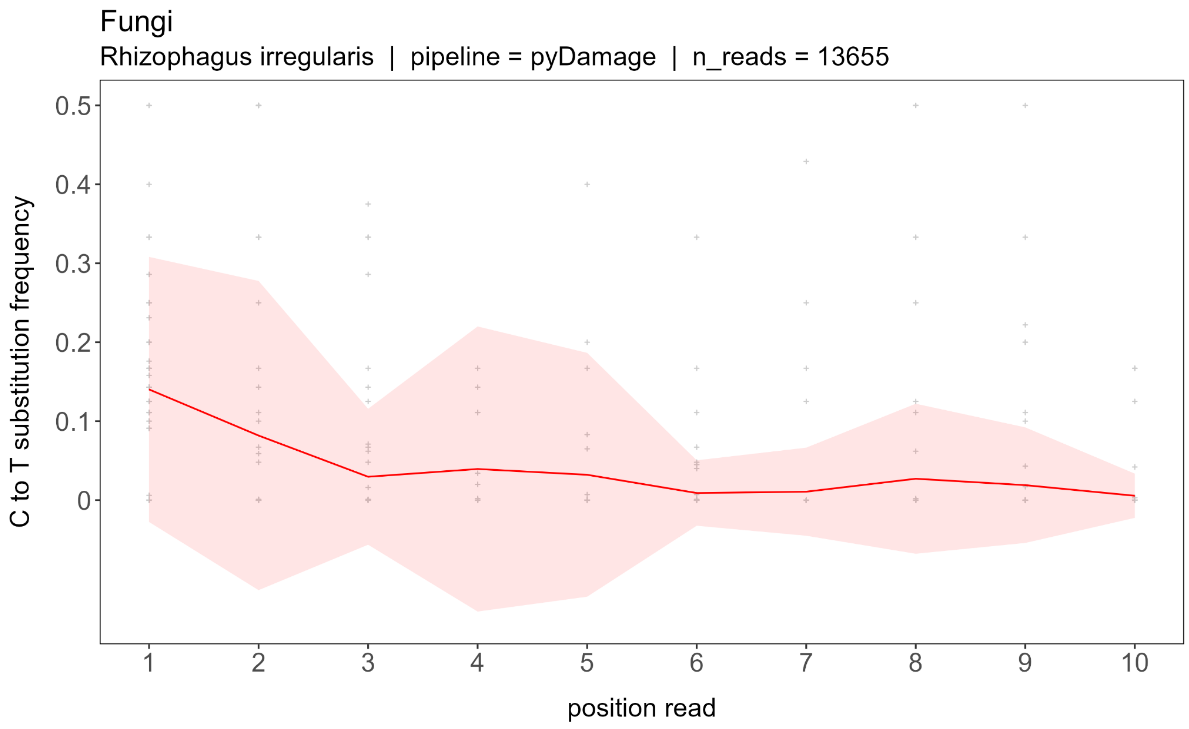

D

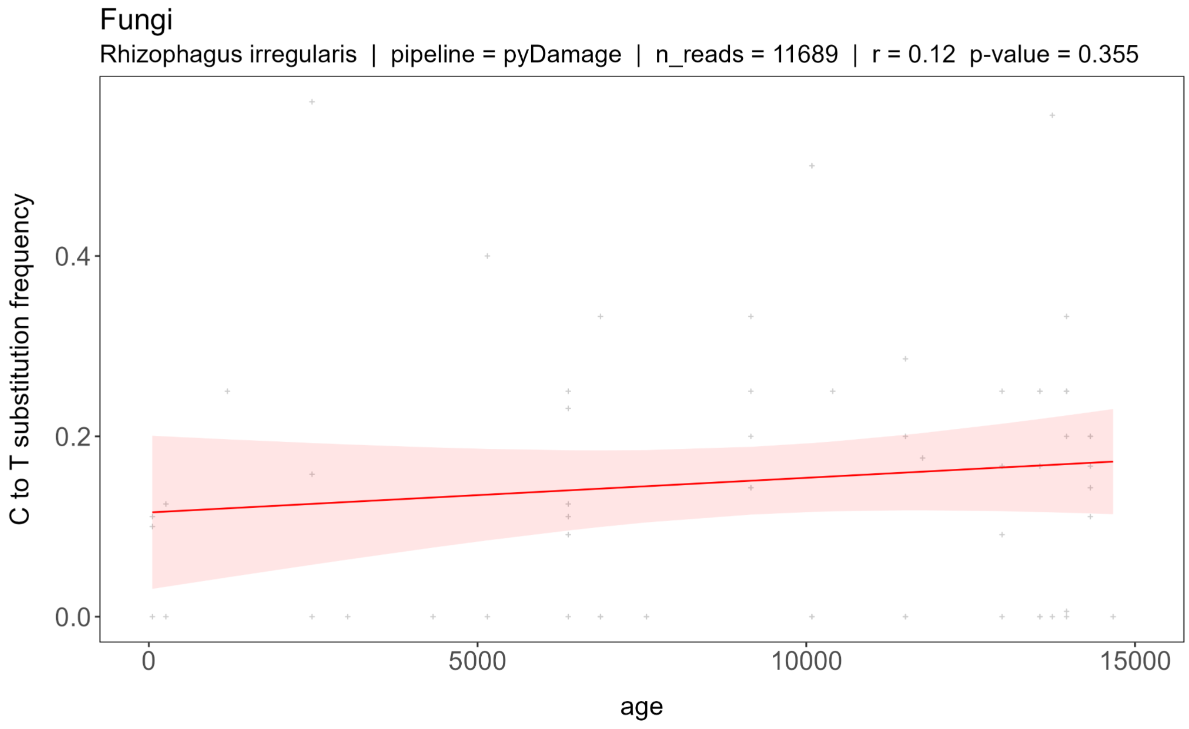

Fig. S11 PyDamage results for Fungi. Data was filtered for a prediction accuracy >=0.6 and a contig length >=1000 bp. **A|** C-to-T substitution frequencies for the first ten read positions for all bacterial contigs from all sample ages. **B|** The C-to-T substitution frequencies for all bacterial contigs at the first position increases significantly with sample age (=<15,000 years). Plot was cut at 0.5 (= 5% of the data points were removed). **C|** C-to-T substitution frequencies for the first ten read positions for all

Rhizophagus irregularis contigs from all sample ages. **D|** The C-to-T substitution frequencies for all *Rhizophagus irregularis* contigs at the first position increases significantly with sample age (=<15,000 years).

Fig. S12

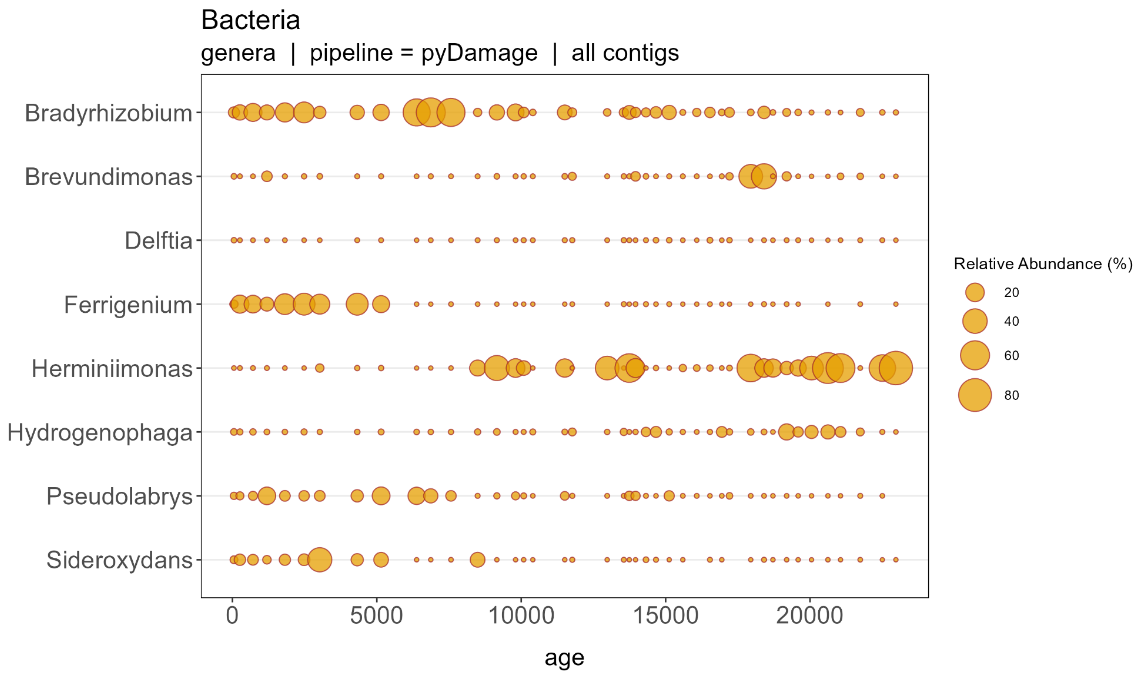
Bacteria
genera | pipeline = pyDamage | all contigs
Bradyrhizobium
Brevundimonas
Delftia
Ferrigenium
Herminiimonas
Hydrogenophaga
Pseudolabrys
Sideroxydans
0
5000
10000
15000
20000
age
Relative Abundance (%)
20
40
60
80

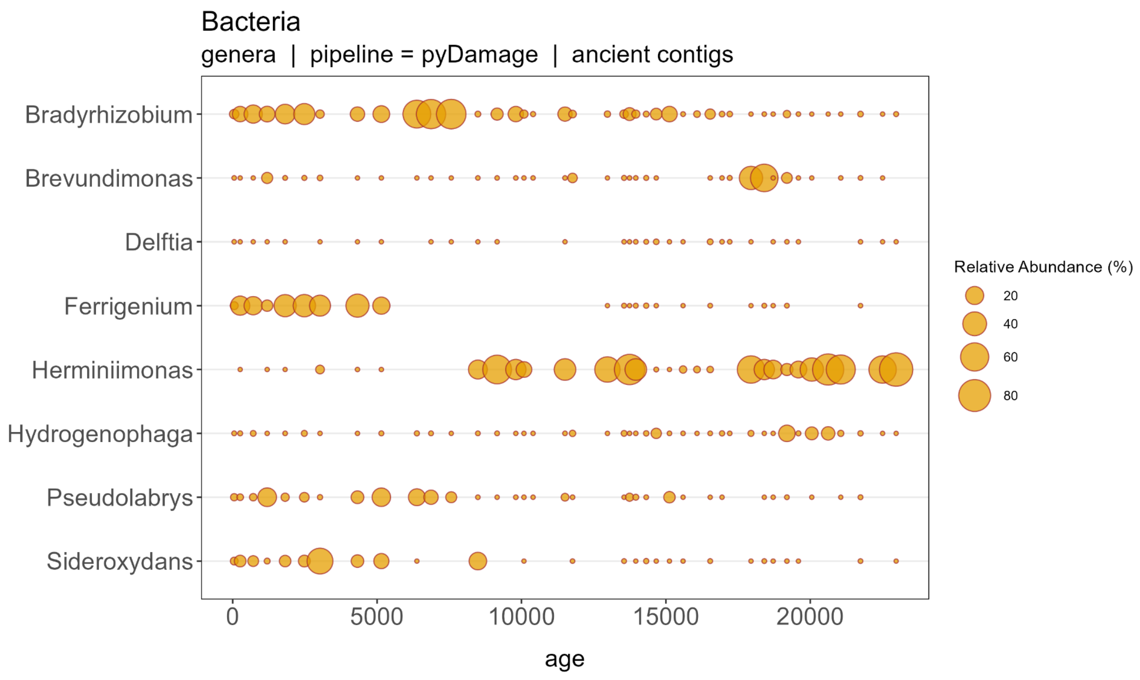
Bacteria
genera | pipeline = pyDamage | ancient contigs
Bradyrhizobium
Brevundimonas
Delftia
Ferrigenium
Herminiimonas
Hydrogenophaga
Pseudolabrys
Sideroxydans
0
5000
10000
15000
20000
age
Relative Abundance (%)
20
40
60
80

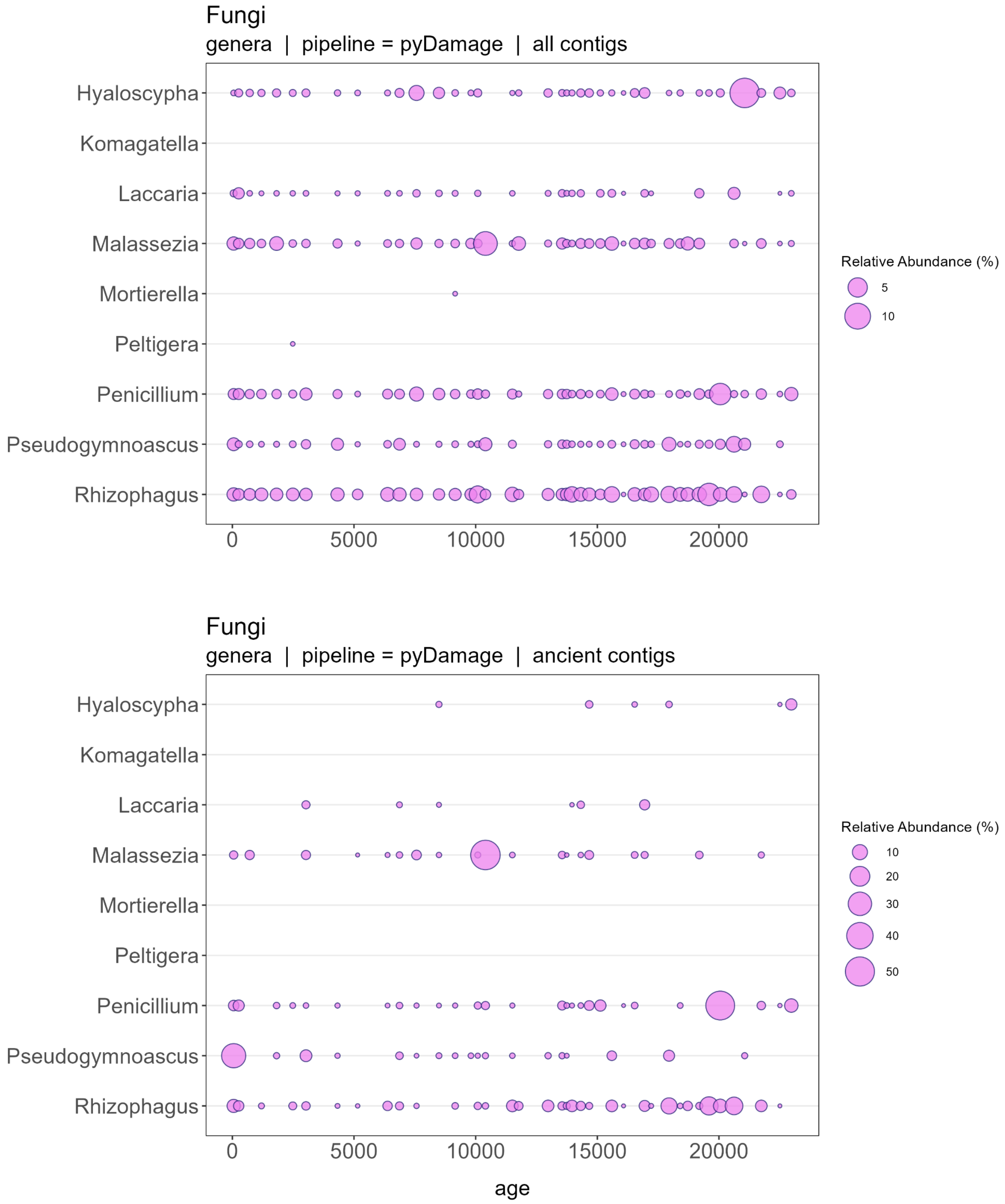

Fig. S12 Comparison of taxonomic temporal distribution of major bacterial and fungal genera extracted from the pyDamage result. The entire unfiltered dataset for A| Bacteria and C| Fungi and the as ancient classified contigs (prediction accuracy >=0.6, contig length>=1000) for B| Bacteria and D| Fungi. Number of reads (related to each contig) were used to estimate the relative abundances of genera in the respective datasets.

Supplementary Tables

Table S1

Procrustes Analysis (comparing PCA ordinations) of the community composition of Bacteria, Fungi and Plants using Kraken2 (taxonomic assignment with k-mers) at the confidence levels 0.8, 0.5 and 0.2. The protest tests (testing the non-randomness (significance) between two configurations derived from procrustes) indicated significantly similar community compositions for all comparisons (p value > 0.05).

		**comparing sample ages**			**comparing taxa**		
	**Comparison of pipeline**	**m12 squared**	**r**	**p**	**m12 squared**	**r**	**p**
**Bacteria**	Kraken nt0.8 vs. Kraken nt0.5	0.028	0.986	0.001	0.377	0.790	0.001
	Kraken nt0.8 vs. Kraken nt0.2	0.099	0.949	0.001	0.480	0.721	0.001
	Kraken nt0.5 vs. Kraken nt0.2	0.030	0.985	0.001	0.620	0.616	0.001
**Fungi**	Kraken nt0.8 vs. Kraken nt0.5	0.245	0.869	0.001	0.419	0.762	0.001
	Kraken nt0.8 vs. Kraken nt0.2	0.321	0.824	0.001	0.672	0.573	0.001
	Kraken nt0.5 vs. Kraken nt0.2	0.097	0.951	0.001	0.399	0.775	0.001
**Plants**	Kraken nt0.8 vs. Kraken nt0.5	0.059	0.970	0.001	0.1333	0.931	0.001
	Kraken nt0.8 vs. Kraken nt0.2	0.091	0.954	0.001	0.2904	0.842	0.001
	Kraken nt0.5 vs. Kraken nt0.2	0.028	0.986	0.001	0.1872	0.902	0.001

Table S2

Procrustes Analysis (comparing PCA ordinations) of the community composition of Bacteria, Fungi and Plants using Kraken2 (taxonomic assignment with k-mers at conf. 0.8), HOPS (taxonomic assignment with malt alignment) and HOLI (taxonomic assignment with ngsLCA alignments). While Kraken and HOPS use the nt database, the HOLI approach uses a customized database (Data S3). The protest tests (testing the non-randomness (significance) between two configurations derived from procrustes) indicated significantly similar community compositions for all comparisons (p value > 0.05).

		comparing sample ages			comparing taxa		
	**Comparison of pipeline**	**m12 squared**	**r**	**p**	**m12 squared**	**r**	**p**
**Bacteria**	Kraken nt0.8 vs. HOPS (default)	0.4025	0.773	0.001	0.7852	0.463	0.001
	Kraken nt0.8 vs. HOLI	0.1108	0.943	0.001	0.6202	0.616	0.001
	HOPS (default) vs. HOLI	0.3723	0.793	0.001	0.3584	0.801	0.001
**Fungi**	Kraken nt0.8 vs. HOPS (default)	0.6122	0.623	0.001	0.7238	0.526	0.001
	Kraken nt0.8 vs. HOLI	0.6769	0.569	0.001	0.8076	0.439	0.001
	HOPS (default) vs. HOLI	0.196	0.897	0.001	0.5747	0.652	0.001
**Plants**	Kraken nt0.8 vs. HOPS (default)	0.09282	0.953	0.001	0.4309	0.754	0.001
	Kraken nt0.8 vs. HOLI	0.2798	0.849	0.001	0.7232	0.526	0.001
	HOPS (default) vs. HOLI	0.3316	0.817	0.001	0.7821	0.467	0.001

Table S3

Mantel test results comparing pairwise distance between samples based on PCA covariance (PC1 and PC2 scores) in a joint ordination space across different taxonomic classification approaches, demonstrating consistency in taxonomic composition relationship between classification approaches.

**Taxonomic groups**	**Classification approaches**	**Mantel_r**	**Mantel_p**
Bacteria	HOLI vs. Kraken	0.96	0.001

Bacteria	HOLI vs. HOPS	0.8	0.001
Bacteria	Kraken vs. HOPS	0.71	0.001
Fungi	HOLI vs. Kraken	0.92	0.001
Fungi	HOLI vs. HOPS	0.91	0.001
Fungi	Kraken vs. HOPS	0.88	0.001
Plants	HOLI vs. Kraken	0.96	0.001
Plants	HOLI vs. HOPS	0.88	0.001
Plants	Kraken vs. HOPS	0.8	0.001

Table S4

Comparison of the classification approaches Kraken2 and HOLI for the different taxonomic groups. The proportion of HOLI reads, when filtering for taxa used from the Kraken output, are given for each taxonomic group.

	HOLI read counts	Kraken nt0.8 read counts	HOLI reads not covered by Kraken2	HOLI reads covered by Kraken2
Plants (genus level)	28,019,782	345,360	450,861 (1.6%)	27,568,921 (99.4%)
Fungi (genus level)	81,146	14,102	3,430 (4.2%)	77,716 (95.8%)
Fungi (phylum level)	113,363	47,889	13,735 (12.1%)	99,628 (87.9%)
Bacteria (genus level)	24,337,984	4,618,183	2,811,179 (11.6%)	21,526,805 (88.4%)

Table S5

Procrustes Analysis (comparing PCA ordinations) of the community composition of Bacteria and Fungi using the pyDamage (taxonomic assignment of contigs with Kraken2 against the nt database with confidence 0.0) and Kraken2 (taxonomic assignment of short reads from the metagenomic shotgun data using the nt database with confidence 0.0). The comparisons include PyDamage (unfiltered data = containing ancient and non-ancient contigs), PyDamage (filtered data with a prediction accuracy of 0.6 and a contig length >=1000bp, using only ancient contigs). The protest tests (testing the non-randomness (significance) between two configurations derived from procrustes) indicated significantly similar community compositions for all comparisons (p value > 0.05).

		comparing sample ages			comparing taxa		
	**Comparison of pipeline**	**m12 squared**	**r**	**p**	**m12 squared**	**r**	**p**
**Bacteria**	Kraken nt0.8 vs. pyDamage	0.7803	0.4687	0.001	0.7746	0.4748	0.001
	pyDamage vs. pyDamage ancient	0.5641	0.6603	0.001	0.5631	0.661	0.001
**Fungi**	Kraken nt0.8 vs. pyDamage	0.8794	0.3473	0.014	0.932	0.2608	0.001
	pyDamage vs. pyDamage ancient	0.7484	0.5016	0.001	0.884	0.3406	0.001

Legends for Data S1 to S3

Data S1 Overview of Lama lake core samples (depth, age and laboratory codes) and raw and filtered results from shotgun metagenomic sequencing

Data S2 Lists for bacteria, fungi and plants identified with the Kraken2 classification and final taxa selection

Data S3 List of reference databases included in the customized reference database for the HOLI pipeline analyses

REFERENCES AND NOTES

1. D. A. Wardle, L. R. Walker, R. D. Bardgett, Ecosystem properties and forest decline in contrasting long-term chronosequences. *Science* **305**, 509–513 (2004).

2. E. A. Davidson, I. A. Janssens, Temperature sensitivity of soil carbon decomposition and feedbacks to climate change. *Nature* **440**, 165–173 (2006).

3. M. Delgado-Baquerizo, P. B. Reich, R. D. Bardgett, D. J. Eldridge, H. Lambers, D. A. Wardle, S. C. Reed, C. Plaza, G. K. Png, S. Neuhauser, A. A. Berhe, S. C. Hart, H.-W. Hu, J.-Z. He, F. Bastida, S. Abades, F. D. Alfaro, N. A. Cutler, A. Gallardo, L. García-Velázquez, P. E. Hayes, Z.-Y. Hseu, C. A. Pérez, F. Santos, C. Siebe, P. Trivedi, B. W. Sullivan, L. Weber-Grullon, M. A. Williams, N. Fierer, The influence of soil age on ecosystem structure and function across biomes. *Nat. Commun.* **11**, 4721 (2020).

4. T. A. Jackson, Weathering, secondary mineral genesis, and soil formation caused by lichens and mosses growing on granitic gneiss in a boreal forest environment. *Geoderma* **251-252**, 78–91 (2015).

5. H. A. Ewing, The influence of substrate on vegetation history and ecosystem development. *Ecology* **83**, 2766–2781 (2002).

6. E. F. Kelly, O. A. Chadwick, T. E. Hilinski, The effect of plants on mineral weathering. *Biogeochemistry* **42**, 21–53 (1998).

7. R. Finlay, H. Wallander, M. Smits, S. Holmstrom, P. van Hees, B. Lian, A. Rosling, The role of fungi in biogenic weathering in boreal forest soils. *Fungal Biol. Rev.* **23**, 101–106 (2009).

8. J. Berthelin, G. Ona-Nguema, S. Stemmler, C. Quantin, M. Abdelmoula, F. Jorand, Bioreduction of ferric species and biogenesis of green rusts in soils. *Comptes Rendus Geosci.* **338**, 447–455 (2006).

9. J. Chen, H.-P. Blume, L. Beyer, Weathering of rocks induced by lichen colonization — a review. *Catena* **39**, 121–146 (2000).

10. P.-E. Courty, M. Buée, A. G. Diedhiou, P. Frey-Klett, F. Le Tacon, F. Rineau, M.-P. Turpault, S. Uroz, J. Garbaye, The role of ectomycorrhizal communities in forest ecosystem processes: New perspectives and emerging concepts. *Soil Biol. Biochem.* **42**, 679–698 (2010).

11. H. Lambers, O. K. Atkin, F. F. Millenaar, "Respiratory patterns in roots in relation to their functioning" in *Plant Roots* (CRC Press, ed. 3, 2002), pp. 810–866.

12. E. Hoffland, T. W. Kuyper, H. Wallander, C. Plassard, A. A. Gorbushina, K. Haselwandter, S. Holmström, R. Landeweert, U. S. Lundström, A. Rosling, R. Sen, M. M. Smits, P. A. van Hees, N. van Breemen, The role of fungi in weathering. *Front. Ecol. Environ.* **2**, 258–264 (2004).

13. M. G. A. Van Der Heijden, R. D. Bardgett, N. M. Van Straalen, The unseen majority: Soil microbes as drivers of plant diversity and productivity in terrestrial ecosystems. *Ecol. Lett.* **11**, 296–310 (2008).

14. M. F. Cotrufo, M. D. Wallenstein, C. M. Boot, K. Denef, E. Paul, The Microbial Efficiency-Matrix Stabilization (MEMS) framework integrates plant litter decomposition with soil organic matter stabilization: Do labile plant inputs form stable soil organic matter? *Glob. Chang. Biol.* **19**, 988–995 (2013).

15. S. Trumbore, Carbon respired by terrestrial ecosystems – Recent progress and challenges. *Glob. Chang. Biol.* **12**, 141–153 (2006).

16. C. Liang, T. C. Balser, Microbial production of recalcitrant organic matter in global soils: Implications for productivity and climate policy. *Nat. Rev. Microbiol.* **9**, 75 (2011).

17. B. D. Lindahl, K. Ihrmark, J. Boberg, S. E. Trumbore, P. Högberg, J. Stenlid, R. D. Finlay, Spatial separation of litter decomposition and mycorrhizal nitrogen uptake in a boreal forest. *New Phytol.* **173**, 611–620 (2007).

18. R. Dixon, D. Kahn, Genetic regulation of biological nitrogen fixation. *Nat. Rev. Microbiol.* **2**, 621–631 (2004).

19. E. George, H. Marschner, I. Jakobsen, Role of arbuscular mycorrhizal fungi in uptake of phosphorus and nitrogen from soil. *Crit. Rev. Biotechnol.* **15**, 257–270 (1995).

20. T. A. M. Pugh, T. Rademacher, S. L. Shafer, J. Steinkamp, J. Barichivich, B. Beckage, V. Haverd, A. Harper, J. Heinke, K. Nishina, A. Rammig, H. Sato, A. Arneth, S. Hantson, T. Hickler, M. Kautz, B. Quesada, B. Smith, K. Thonicke, Understanding the uncertainty in global forest carbon turnover. *Biogeosciences* **17**, 3961–3989 (2020).

21. U. S. Lundström, N. van Breemen, D. Bain, The podzolization process. A review. *Geoderma* **94**, 91–107 (2000).

22. L. Wiklander, A. Andersson, The replacing efficiency of hydrogen ion in relation to base saturation and pH. *Geoderma* **7**, 159–165 (1972).

23. D. Sauer, H. Sponagel, M. Sommer, L. Giani, R. Jahn, K. Stahr, Podzol: Soil of the year 2007. A review on its genesis, occurrence, and functions. *J. Plant Nutr. Soil Sci.* **170**, 581–597 (2007).

24. M. E. D'Amico, M. Freppaz, G. Filippa, E. Zanini, Vegetation influence on soil formation rate in a proglacial chronosequence (Lys Glacier, NW Italian Alps). *Catena* **113**, 122–137 (2014).

25. C. Giguet-Covex, G. F. Ficetola, K. Walsh, J. Poulenard, M. Bajard, L. Fouinat, P. Sabatier, L. Gielly, E. Messager, A. L. Develle, F. David, P. Taberlet, E. Brisset, F. Guiter, R. Sinet, F. Arnaud, New insights on lake sediment DNA from the catchment: Importance of taphonomic and analytical issues on the record quality. *Sci. Rep.* **9**, 14676 (2019).

26. E. Capo, C. Giguet-Covex, A. Rouillard, K. Nota, P. D. Heintzman, A. Vuillemin, D. Ariztegui, F. Arnaud, S. Belle, S. Bertilsson, C. Bigler, R. Bindler, A. G. Brown, C. L. Clarke, S. E. Crump, D. Debroas, G. Englund, G. F. Ficetola, R. E. Garner, J. Gauthier, I. Gregory-Eaves, L. Heinecke, U. Herzschuh, A. Ibrahim, V. Kisand, K. H. Kjær, Y. Lammers, J. Littlefair, E. Messager, M.-E. Monchamp, F. Olajos, W. Orsi, M. W. Pedersen, D. P. Rijal, J. Rydberg, T. Spanbauer, K. R. Stoof-Leichsenring, P. Taberlet, L. Talas, C. Thomas, D. A. Walsh, Y. Wang, E. Willerslev, A. van Woerkom, H. H. Zimmermann, M. J. L. Coolen, L. S.

Epp, I. Domaizon, I. G. Alsos, L. Parducci, Lake sedimentary DNA research on past terrestrial and aquatic biodiversity: Overview and recommendations. *Quaternary* **4**, 6 (2021).

27. L. Parducci, I. G. Alsos, P. Unneberg, M. W. Pedersen, L. Han, Y. Lammers, J. S. Salonen, M. M. Väliranta, T. Slotte, B. Wohlfarth, Shotgun environmental DNA, pollen, and macrofossil analysis of lateglacial lake sediments from southern Sweden. *Front. Ecol. Evol.* **7**, 189 (2019).

28. L. Talas, N. Stivrins, S. Veski, L. Tedersoo, V. Kisand, Sedimentary ancient DNA (sedaDNA) reveals fungal diversity and environmental drivers of community changes throughout the Holocene in the present Boreal Lake Lielais Svētiņu (Eastern Latvia). *Microorganisms* **9**, 719 (2021).

29. K. H. Kjær, M. Winther Pedersen, B. De Sanctis, B. De Cahsan, T. S. Korneliussen, C. S. Michelsen, K. K. Sand, S. Jelavić, A. H. Ruter, A. M. A. Schmidt, K. K. Kjeldsen, A. S. Tesakov, I. Snowball, J. C. Gosse, I. G. Alsos, Y. Wang, C. Dockter, M. Rasmussen, M. E. Jørgensen, B. Skadhauge, A. Prohaska, J. Å. Kristensen, M. Bjerager, M. E. Allentoft, E. Coissac, A. Rouillard, A. Simakova, A. Fernandez-Guerra, C. Bowler, M. Macias-Fauria, L. Vinner, J. J. Welch, A. J. Hidy, M. Sikora, M. J. Collins, R. Durbin, N. K. Larsen, E. Willerslev, A 2-million-year-old ecosystem in Greenland uncovered by environmental DNA. *Nature* **612**, 283–291 (2022).

30. S. Liu, K. R. Stoof-Leichsenring, L. Harms, L. Schulte, S. Mischke, S. Kruse, C. Zhang, U. Herzschuh, Tibetan terrestrial and aquatic ecosystems collapsed with cryosphere loss inferred from sedimentary ancient metagenomics. *Sci. Adv.* **10**, eadn8490 (2024).

31. B. Niemeyer, L. S. Epp, K. R. Stoof-Leichsenring, L. A. Pestryakova, U. Herzschuh, A comparison of sedimentary DNA and pollen from lake sediments in recording vegetation composition at the Siberian treeline. *Mol. Ecol. Resour.* **17**, e46–e62 (2017).

32. M. E. Edwards, I. G. Alsos, N. Yoccoz, E. Coissac, T. Goslar, L. Gielly, J. Haile, C. T. Langdon, A. Tribsch, H. A. Binney, H. von Stedingk, P. Taberlet, Metabarcoding of modern soil DNA gives a highly local vegetation signal in Svalbard tundra. *Holocene* **28**, 2006–2016 (2018).

33. P. Sjögren, M. E. Edwards, L. Gielly, C. T. Langdon, I. W. Croudace, M. K. F. Merkel, T. Fonville, I. G. Alsos, Lake sedimentary DNA accurately records 20th Century introductions of exotic conifers in Scotland. *New Phytol.* **213**, 929–941 (2017).

34. I. G. Alsos, Y. Lammers, N. G. Yoccoz, T. Jørgensen, P. Sjögren, L. Gielly, M. E. Edwards, Plant DNA metabarcoding of lake sediments: How does it represent the contemporary vegetation. *PLOS ONE* **13**, e0195403 (2018).

35. B. von Hippel, "Long-term bacteria-fungi-plant associations in permafrost soils inferred from palaeometagenomics," thesis, Universität Potsdam (2024).

36. B. von Hippel, K. R. Stoof-Leichsenring, L. Schulte, P. Seeber, L. S. Epp, B. K. Biskaborn, B. Diekmann, M. Melles, L. Pestryakova, U. Herzschuh, Long-term fungus–plant covariation from multi-site sedimentary ancient DNA metabarcoding. *Quat. Sci. Rev.* **295**, 107758 (2022).

37. A. A. Andreev, P. E. Tarasov, V. A. Klimanov, M. Melles, O. M. Lisitsyna, H.-W. Hubberten, Vegetation and climate changes around the Lama Lake, Taymyr Peninsula, Russia during the Late Pleistocene and Holocene. *Quat. Int.* **122**, 69–84 (2004).

38. A. Zumsteg, J. Luster, H. Göransson, R. H. Smittenberg, I. Brunner, S. M. Bernasconi, J. Zeyer, B. Frey, Bacterial, archaeal and fungal succession in the forefield of a receding glacier. *Microb. Ecol.* **63**, 552–564 (2012).

39. M. V. Korneikova, Comparative analysis of the number and structure of the complexes of microscopic fungi in tundra and taiga soils in the north of the Kola Peninsula. *Eurasian Soil Sci.* **51**, 89–95 (2018).

40. D. Muller, D. D. Simeonova, P. Riegel, S. Mangenot, S. Koechler, D. Lièvremont, P. N. Bertin, M.-C. Lett, *Herminiimonas arsenicoxydans* sp. nov., a metalloresistant bacterium. *Int. J. Syst. Evol. Microbiol.* **56**, 1765–1769 (2006).

41. C. Kabala, Ł. Chachulski, B. Gądek, B. Korabiewski, M. Mętrak, M. Suska-Malawska, Soil development and spatial differentiation in a glacial river valley under cold and extremely arid climate of East Pamir Mountains. *Sci. Total Environ.* **758**, 144308 (2021).

42. R. E. Garner, I. Gregory-Eaves, D. A. Walsh, Sediment metagenomes as time capsules of lake microbiomes. *mSphere* **5**, e00512-20 (2020).

43. H. H. Zimmermann, K. R. Stoof-Leichsenring, V. Dinkel, L. Harms, L. Schulte, M.-T. Hütt, D. Nürnberg, R. Tiedemann, U. Herzschuh, Marine ecosystem shifts with deglacial sea-ice loss inferred from ancient DNA shotgun sequencing. *Nat. Commun.* **14**, 1650 (2023).

44. X. Han, J. Tolu, L. Deng, A. Fiskal, C. J. Schubert, L. H. E. Winkel, M. A. Lever, Long-term preservation of biomolecules in lake sediments: Potential importance of physical shielding by recalcitrant cell walls. *PNAS Nexus* **1**, pgac076 (2022).

45. V. Pérez, Y. Liu, M. B. Hengst, L. S. Weyrich, A case study for the recovery of authentic microbial ancient DNA from soil samples. *Microorganisms* **10**, 1623 (2022).

46. A. D. Williams, V. W. Leung, J. W. Tang, N. Hidekazu, N. Suzuki, A. C. Clarke, D. A. Pearce, T. T.-Y. Lam, Ancient environmental microbiomes and the cryosphere. *Trends Microbiol.* **33**, 233–249 (2025).

47. J. R. Deslippe, M. Hartmann, S. W. Simard, W. W. Mohn, Long-term warming alters the composition of Arctic soil microbial communities. *FEMS Microbiol. Ecol.* **82**, 303–315 (2012).

48. M. Krishna, S. Gupta, M. Delgado-Baquerizo, E. Morriën, S. C. Garkoti, R. Chaturvedi, S. Ahmad, Successional trajectory of bacterial communities in soil are shaped by plant-driven changes during secondary succession. *Sci. Rep.* **10**, 11461 (2020).

49. N. Schmidt, M. Bölter, Fungal and bacterial biomass in tundra soils along an arctic transect from Taimyr Peninsula, central Siberia. *Polar Biol.* **25**, 871–877 (2002).

50. M. P. Berg, J. P. Kniese, H. A. Verhoef, Dynamics and stratification of bacteria and fungi in the organic layers of a scots pine forest soil. *Biol. Fertil. Soils* **26**, 313–322 (1998).

51. A. Franzetti, F. Pittino, I. Gandolfi, R. S. Azzoni, G. Diolaiuti, C. Smiraglia, M. Pelfini, C. Compostella, B. Turchetti, P. Buzzini, R. Ambrosini, Early ecological succession patterns of bacterial, fungal and plant communities along a chronosequence in a recently deglaciated area of the Italian Alps. *FEMS Microbiol. Ecol.* **96**, fiaa165 (2020).

52. Y. Jiang, Y. Lei, Y. Yang, H. Korpelainen, Ü. Niinemets, C. Li, Divergent assemblage patterns and driving forces for bacterial and fungal communities along a glacier forefield chronosequence. *Soil Biol. Biochem.* **118**, 207–216 (2018).

53. J. Voříšková, B. Elberling, A. Priemé, Fast response of fungal and prokaryotic communities to climate change manipulation in two contrasting tundra soils. *Environ. Microbiome* **14**, 6 (2019).

54. S. K. Liu, C. Han, J. M. Liu, H. Li, Hydrothermal decomposition of potassium feldspar under alkaline conditions. *RSC Adv.* **5**, 93301–93309 (2015).

55. S. A. Vyse, U. Herzschuh, A. A. Andreev, L. A. Pestryakova, B. Diekmann, S. J. Armitage, B. K. Biskaborn, Geochemical and sedimentological responses of arctic glacial Lake Ilirney, chukotka (far east Russia) to palaeoenvironmental change since □51.8 ka BP. *Quat. Sci. Rev.* **247**, 106607 (2020).

56. C. B. Zambell, J. M. Adams, M. L. Gorring, D. W. Schwartzman, Effect of lichen colonization on chemical weathering of hornblende granite as estimated by aqueous elemental flux. *Chem. Geol.* **291**, 166–174 (2012).

57. Y. N. Vodyanitskii, Iron hydroxides in soils: A review of publications. *Eurasian Soil Sci.* **43**, 1244–1254 (2010).

58. H. D. Pedersen, D. Postma, R. Jakobsen, Release of arsenic associated with the reduction and transformation of iron oxides. *Geochim. Cosmochim. Acta* **70**, 4116–4129 (2006).

59. S. I. Lang, J. H. C. Cornelissen, G. R. Shaver, M. Ahrens, T. V. Callaghan, U. Molau, C. J. F. Ter Braak, A. Hölzer, R. Aerts, Arctic warming on two continents has consistent negative

effects on lichen diversity and mixed effects on bryophyte diversity. *Glob. Chang. Biol.* **18**, 1096–1107 (2012).

60. R. Landeweert, E. Hoffland, R. D. Finlay, T. W. Kuyper, N. van Breemen, Linking plants to rocks: Ectomycorrhizal fungi mobilize nutrients from minerals. *Trends Ecol. Evol.* **16**, 248–254 (2001).

61. S. R. Law, A. R. Serrano, Y. Daguerre, J. Sundh, A. N. Schneider, Z. R. Stangl, D. Castro, M. Grabherr, T. Näsholm, N. R. Street, V. Hurry, Metatranscriptomics captures dynamic shifts in mycorrhizal coordination in boreal forests. *Proc. Natl. Acad. Sci. U.S.A.* **119**, e2118852119 (2022).

62. M. C. Brundrett, L. Tedersoo, Evolutionary history of mycorrhizal symbioses and global host plant diversity. *New Phytol.* **220**, 1108–1115 (2018).

63. Z. Zhou, T. Hogetsu, Subterranean community structure of ectomycorrhizal fungi under *Suillus grevillei* sporocarps in a *Larix kaempferi* forest. *New Phytol.* **154**, 529–539 (2002).

64. N. Praeg, P. Illmer, Microbial community composition in the rhizosphere of *Larix decidua* under different light regimes with additional focus on methane cycling microorganisms. *Sci. Rep.* **10**, 22324 (2020).

65. Z. Balogh-Brunstad, C. Kent Keller, J. Thomas Dickinson, F. Stevens, C. Y. Li, B. T. Bormann, Biotite weathering and nutrient uptake by ectomycorrhizal fungus, Suillus tomentosus, in liquid-culture experiments. *Geochim. Cosmochim. Acta* **72**, 2601–2618 (2008).

66. G. M. Mueller, Systematics of Laccaria (Agaricales) in the continental United States and Canada, with discussions on extralimital taxa and descriptions of extant types. *Fieldiana Bot.* **30**, 158 (1992).

67. B. Münzenberger, I. Kottke, F. Oberwinkler, Reduction of phenolics in mycorrhizas of *Larix decidua* Mill. *Tree Physiol.* **15**, 191–196 (1995).

68. J. Fehrer, M. Réblová, V. Bambasová, M. Vohník, The root-symbiotic *Rhizoscyphus ericae* aggregate and *Hyaloscypha* (*Leotiomycetes*) are congeneric: Phylogenetic and experimental evidence. *Stud. Mycol.* **92**, 195–225 (2019).

69. R. E. Hewitt, H. D. Alexander, B. Izbicki, M. M. Loranty, S. M. Natali, X. J. Walker, M. C. Mack, Increasing tree density accelerates stand-level nitrogen cycling at the taiga–tundra ecotone in northeastern Siberia. *Ecosphere* **13**, e4175 (2022).

70. W. Gao, W. Sun, X. Xu, Permafrost response to temperature rise in carbon and nutrient cycling: Effects from habitat-specific conditions and factors of warming. *Ecol. Evol.* **11**, 16021–16033 (2021).

71. D. N. Gabov, V. A. Beznosikov, Polycyclic aromatic hydrocarbons in tundra soils of the Komi Republic. *Eurasian Soil Sci.* **47**, 18–25 (2014).

72. S. Murayama, Y. Sugiura, "Origin of soil polysaccharides, and ectomycorrhizal fungal sclerotia as sources of forest soil polysaccharides" in *Sclerotia Grains in Soils: A New Perspective from Pedosclerotiology*, *Progress in Soil Science*, M. Watanabe, Ed. (Springer, 2021), pp. 91–117.

73. M.-C. Nilsson, D. A. Wardle, T. H. DeLuca, Belowground and aboveground consequences of interactions between live plant species mixtures and dead organic substrate mixtures. *Oikos* **117**, 439–449 (2008).

74. A. Clocchiatti, S. E. Hannula, M. van den Berg, M. P. J. Hundscheid, W. de Boer, Evaluation of phenolic root exudates as stimulants of saptrophic fungi in the rhizosphere. *Front. Microbiol.* **12**, 644046 (2021).

75. J. B. Brant, E. W. Sulzman, D. D. Myrold, Microbial community utilization of added carbon substrates in response to long-term carbon input manipulation. *Soil Biol. Biochem.* **38**, 2219–2232 (2006).

76. K. Rousk, P. L. Sorensen, A. Michelsen, What drives biological nitrogen fixation in high arctic tundra: Moisture or temperature? *Ecosphere* **9**, e02117 (2018).

77. M. J. Gundale, M. Nilsson, S. Bansal, A. Jäderlund, The interactive effects of temperature and light on biological nitrogen fixation in boreal forests. *New Phytol.* **194**, 453–463 (2012).

78. K. J. Stewart, P. Grogan, D. S. Coxson, S. D. Siciliano, Topography as a key factor driving atmospheric nitrogen exchanges in arctic terrestrial ecosystems. *Soil Biol. Biochem.* **70**, 96–112 (2014).

79. M. Chiwa, A. Crossley, L. J. Sheppard, H. Sakugawa, J. N. Cape, Throughfall chemistry and canopy interactions in a Sitka spruce plantation sprayed with six different simulated polluted mist treatments. *Environ. Pollut.* **127**, 57–64 (2004).

80. D. Bryan Dail, D. Y. Hollinger, E. A. Davidson, I. Fernandez, H. C. Sievering, N. A. Scott, E. Gaige, Distribution of nitrogen-15 tracers applied to the canopy of a mature spruce-hemlock stand, Howland, Maine, USA. *Oecologia* **160**, 589–599 (2009).

81. H. Sievering, T. Tomaszewski, J. Torizzo, Canopy uptake of atmospheric N deposition at a conifer forest: Part I -canopy N budget, photosynthetic efficiency and net ecosystem exchange. *Tellus B Chem. Phys. Meteorol.* **59**, 483–492 (2007).

82. J. P. Sparks, Ecological ramifications of the direct foliar uptake of nitrogen. *Oecologia* **159**, 1–13 (2009).

83. R. G. McLaren, K. C. Cameron, R. G. McLaren, K. C. Cameron, *Soil Science: Sustainable Production and Environmental Protection* (Oxford Univ. Press, 1996).

84. J. Feng, C. Wang, J. Lei, Y. Yang, Q. Yan, X. Zhou, X. Tao, D. Ning, M. M. Yuan, Y. Qin, Z. J. Shi, X. Guo, Z. He, J. D. Van Nostrand, L. Wu, R. G. Bracho-Garillo, C. R. Penton, J. R. Cole, K. T. Konstantinidis, Y. Luo, E. A. G. Schuur, J. M. Tiedje, J. Zhou, Warming-induced permafrost thaw exacerbates tundra soil carbon decomposition mediated by microbial community. *Microbiome* **8**, 3 (2020).

85. D. L. Jones, K. Kielland, Soil amino acid turnover dominates the nitrogen flux in permafrost-dominated taiga forest soils. *Soil Biol. Biochem.* **34**, 209–219 (2002).

86. O. Priha, A. Smolander, Nitrogen transformations in soil under *Pinus sylvestris*, *Picea abies* and *Betula pendula* at two forest sites. *Soil Biol. Biochem.* **31**, 965–977 (1999).

87. T. W. Berger, P. Berger, Greater accumulation of litter in spruce (*Picea abies*) compared to beech (*Fagus sylvatica*) stands is not a consequence of the inherent recalcitrance of needles. *Plant Soil* **358**, 349–369 (2012).

88. E. Desie, K. Vancampenhout, L. van den Berg, B. Nyssen, M. Weijters, J. den Ouden, B. Muys, Litter share and clay content determine soil restoration effects of rich litter tree species in forests on acidified sandy soils. *For. Ecol. Manage.* **474**, 118377 (2020).

89. C. Kim, H.-C. An, H.-S. Cho, G.-C. Choo, Base cation fluxes and release by needle litter in three adjacent coniferous plantations. *For. Sci. Technol.* **9**, 225–228 (2013).

90. X. Zhang, W. Liu, G. Zhang, L. Jiang, X. Han, Mechanisms of soil acidification reducing bacterial diversity. *Soil Biol. Biochem.* **81**, 275–281 (2015).

91. J. Rousk, E. Bååth, Growth of saprotrophic fungi and bacteria in soil. *FEMS Microbiol. Ecol.* **78**, 17–30 (2011).

92. R. H. Bares, M. K. Wali, Chemical relations and litter production of Picea mariana and Larix laricina stands on an alkaline peatland in Northern Minnesota. *Vegetatio* **40**, 79–94 (1979).

93. S. Gislason, W. S. Broecker, E. Gunnlaugsson, S. Snæbjörnsdóttir, K. G. Mesfin, H. A. Alfredsson, E. S. Aradottir, B. Sigfusson, I. Gunnarsson, M. Stute, J. M. Matter, M. T. Arnarson, I. M. Galeczka, S. Gudbrandsson, G. Stockmann, D. Wolff-Boenisch, A. Stefansson, E. Ragnheidardottir, T. Flaathen, A. P. Gysi, E. Oelkers, Rapid solubility and mineral storage of CO_2 in basalt. *Energy Procedia* **63**, 4561–4574 (2014).

94. S. Ó. Snæbjörnsdóttir, B. Sigfússon, C. Marieni, D. Goldberg, S. R. Gislason, E. H. Oelkers, Carbon dioxide storage through mineral carbonation. *Nat. Rev. Earth Environ.* **1**, 90–102 (2020).

95. D. S. Goll, P. Ciais, T. Amann, W. Buermann, J. Chang, S. Eker, J. Hartmann, I. Janssens, W. Li, M. Obersteiner, J. Penuelas, K. Tanaka, S. Vicca, Potential CO_2 removal from enhanced weathering by ecosystem responses to powdered rock. *Nat. Geosci.* **14**, 545–549 (2021).

96. Russian Institute of Hydrometeorological Information, World Data Center (2021). http://meteo.ru/.

97. I. Croudace, A. Rindby, R. G. Rothwell, ITRAX: Description and evaluation of a new multi-function X-ray core scanner. *Geol. Soc. Lond. Spec. Publ.* **267**, 51–63 (2006).

98. S. Shala, K. F. Helmens, K. N. Jansson, M. E. Kylander, J. Risberg, L. Löwemark, Palaeoenvironmental record of glacial lake evolution during the early Holocene at Sokli, NE Finland. *Boreas* **43**, 362–376 (2014).

99. M.-T. Gansauge, T. Gerber, I. Glocke, P. Korlevic, L. Lippik, S. Nagel, L. M. Riehl, A. Schmidt, M. Meyer, Single-stranded DNA library preparation from highly degraded DNA using T4 DNA ligase. *Nucleic Acids Res.* **45**, e79 (2017).

100. L. Schulte, N. Bernhardt, K. Stoof-Leichsenring, H. H. Zimmermann, L. A. Pestryakova, L. S. Epp, U. Herzschuh, Hybridization capture of larch (*Larix* Mill.) chloroplast genomes from sedimentary ancient DNA reveals past changes of Siberian forest. *Mol. Ecol. Resour.* **21**, 801–815 (2021).

101. S. Andrews, "FastQC: A quality control tool for high throughput sequence data" (2010); https://www.bioinformatics.babraham.ac.uk/projects/fastqc/.

102. S. Chen, Y. Zhou, Y. Chen, J. Gu, fastp: An ultra-fast all-in-one FASTQ preprocessor. *Bioinformatics* **34**, i884–i890 (2018).

103. I. Malik, Ł. Pawlik, A. Ślęzak, M. Wistuba, A study of the wood anatomy of *Picea abies* roots and their role in biomechanical weathering of rock cracks. *Catena* **173**, 264–275 (2019).

104. R. Hübler, F. M. Key, C. Warinner, K. I. Bos, J. Krause, A. Herbig, HOPS: Automated detection and authentication of pathogen DNA in archaeological remains. *Genome Biol.* **20**, 280 (2019).

105. M. W. Pedersen, A. Ruter, C. Schweger, H. Friebe, R. A. Staff, K. K. Kjeldsen, M. L. Z. Mendoza, A. B. Beaudoin, C. Zutter, N. K. Larsen, B. A. Potter, R. Nielsen, R. A. Rainville, L. Orlando, D. J. Meltzer, K. H. Kjær, E. Willerslev, Postglacial viability and colonization in North America's ice-free corridor. *Nature* **537**, 45–49 (2016).

106. M. Borry, A. Hübner, A. B. Rohrlach, C. Warinner, PyDamage: Automated ancient damage identification and estimation for contigs in ancient DNA de novo assembly. *PeerJ* **9**, e11845 (2021).

107. J. D. Shakun, P. U. Clark, F. He, S. A. Marcott, A. C. Mix, Z. Liu, B. Otto-Bliesner, A. Schmittner, E. Bard, Global warming preceded by increasing carbon dioxide concentrations during the last deglaciation. *Nature* **484**, 49–54 (2012).

108. S. A. Marcott, J. D. Shakun, P. U. Clark, A. C. Mix, A reconstruction of regional and global temperature for the past 11,300 years. *Science* **339**, 1198–1201 (2013).

109. H. Jónsson, A. Ginolhac, M. Schubert, P. L. F. Johnson, L. Orlando, mapDamage2.0: Fast approximate Bayesian estimates of ancient DNA damage parameters. *Bioinformatics* **29**, 1682–1684 (2013).

110. J. Courtin, A. Perfumo, A. A. Andreev, T. Opel, K. R. Stoof-Leichsenring, M. E. Edwards, J. B. Murton, U. Herzschuh, Pleistocene glacial and interglacial ecosystems inferred from ancient DNA analyses of permafrost sediments from Batagay megaslump, East Siberia. *Environ. DNA* **4**, 1265–1283 (2022).

111. S. E. Crump, Sedimentary ancient DNA as a tool in paleoecology. *Nat. Rev. Earth Environ.* **2**, 229–229 (2021).

112. L. Kistler, R. Ware, O. Smith, M. Collins, R. G. Allaby, A new model for ancient DNA decay based on paleogenomic meta-analysis. *Nucleic Acids Res.* **45**, 6310–6320 (2017).

113. B. Bushnell, "BBMap: A fast, accurate, splice-aware aligner" (2014); https://sourceforge.net/projects/bbmap.

114. D. Li, R. Luo, C. Liu, C. Leung, H. Ting, K. Sadakane, H. Yamashita, T. Lam, MEGAHIT v1.0: A fast and scalable metagenome assembler driven by advanced methodologies and community practices. *Methods* **102**, 3–11 (2016).

115. N. Oskolkov, A. Sandionigi, A. Gotherstrom, F. Canini, B. Turchetti, L. Zucconi, T. Mimmo, P. Buzzini, L. Borruso, Unravelling the ancient fungal DNA from the Iceman's gut. *BMC Genomics* **25**, 1225 (2024).

116. K. Frey, B. Pucker, Animal, fungi, and plant genome sequences harbor different non-canonical splice sites. *Cells* **9**, 458 (2020).

117. S. Mehrotra, V. Goyal, Repetitive sequences in plant nuclear DNA: Types, distribution, evolution and function. *Genomics Proteomics Bioinformatics* **12**, 164–171 (2014).

118. J. Wöstemeyer, A. Kreibich, Repetitive DNA elements in fungi (Mycota): Impact on genomic architecture and evolution. *Curr. Genet.* **41**, 189–198 (2002).

119. B. A. Nimeth, S. Riegler, M. Kalyna, Alternative Splicing and DNA damage response in plants. *Front. Plant Sci.* **11**, 91 (2020).

120. R. H. Nilsson, C. Wurzbacher, M. Bahram, V. R. M. Coimbra, E. Larsson, L. Tedersoo, J. Eriksson, C. Duarte, S. Svantesson, M. Sanchez-Garcia, M. K. Ryberg, E. Kristiansson, K. Abarenkov, Top 50 most wanted fungi. *MycoKeys* **12**, 29–40 (2016).

121. P. A. Seeber, B. von Hippel, H. Kauserud, U. Lober, K. R. Stoof-Leichsenring, U. Herzschuh, L. S. Epp, Evaluation of lake sedimentary ancient DNA metabarcoding to assess fungal biodiversity in Arctic paleoecosystems. *Environ. DNA* **4**, 1150–1163 (2022).

122. E. Capo, M. Monchamp, M. J. L. Coolen, I. Domaizon, L. Armbrecht, S. Bertilsson, Environmental paleomicrobiology: Using DNA preserved in aquatic sediments to its full potential. *Environ. Microbiol.* **24**, 2201–2209 (2022).
